# Site-Specific N- and O-Glycosylation Analysis of Human Plasma Fibronectin

**DOI:** 10.3389/fchem.2021.691217

**Published:** 2021-06-15

**Authors:** Ding Liu, Shuaishuai Wang, Junping Zhang, Weidong Xiao, Carol H. Miao, Barbara A. Konkle, Xiu-Feng Wan, Lei Li

**Affiliations:** ^1^ Department of Chemistry, Georgia State University, Atlanta, GA, United States; ^2^ School of Medicine, Indiana University, Indianapolis, IN, United States; ^3^ Center for Immunity and Immunotherapies, Seattle Children’s Research Institute, Seattle, WA, United States; ^4^ Bloodworks Northwest, Seattle, WA, United States; ^5^ Center for Influenza and Emerging Infectious Diseases, University of Missouri, Columbia, MO, United States; ^6^ Department of Molecular Microbiology and Immunology, School of Medicine, University of Missouri, Columbia, MO, United States; ^7^ Bond Life Sciences Center, University of Missouri, Columbia, MO, United States; ^8^ Department of Electrical Engineering & Computer Science, College of Engineering, University of Missouri, Columbia, MO, United States

**Keywords:** fibronectin, glycosylation, mass spectrometer, operator, stepped normalized collision energy

## Abstract

Human plasma fibronectin is an adhesive protein that plays a crucial role in wound healing. Many studies had indicated that glycans might mediate the expression and functions of fibronectin, yet a comprehensive understanding of its glycosylation is still missing. Here, we performed a comprehensive N- and O-glycosylation mapping of human plasma fibronectin and quantified the occurrence of each glycoform in a site-specific manner. Intact N-glycopeptides were enriched by zwitterionic hydrophilic interaction chromatography, and N-glycosite sites were localized by the ^18^O-labeling method. O-glycopeptide enrichment and O-glycosite identification were achieved by an enzyme-assisted site-specific extraction method. An RP–LC–MS/MS system functionalized with collision-induced dissociation and stepped normalized collision energy (sNCE)-HCD tandem mass was applied to analyze the glycoforms of fibronectin. A total of 6 N-glycosites and 53 O-glycosites were identified, which were occupied by 38 N-glycoforms and 16 O-glycoforms, respectively. Furthermore, 77.31% of N-glycans were sialylated, and O-glycosylation was dominated by the sialyl-T antigen. These site-specific glycosylation patterns on human fibronectin can facilitate functional analyses of fibronectin and therapeutics development.

## Introduction

As a major post-translational modification (PTM) of proteins, glycosylation has been reported to play critical roles in protein folding, stability, macromolecular interactions, functions, and activity (Mitra et al., 2003; Shental-Bechor and Levy, 2008). Alteration of glycosylation can also affect the immunogenicity of therapeutic proteins, as demonstrated in the case of interferon and others (Kuriakose et al., 2016). N-linked glycosylation and O-linked glycosylation are two major types of protein glycosylation (Varki et al., 2015). The most common O-linked glycosylation refers to the attachment of an α-linked GalNAc residue (or extended structures) to serine (Ser) or threonine (Thr) residues of protein by an O-glycosidic bond, whereas N-linked glycosylation refers to the attachment of a β-linked N-glycan to asparagine (Asn) residues within a consensus peptide sequence of Asn-Xxx (not Pro)-Ser/Thr *via* an N-glycosidic bond.

Human plasma fibronectin is a large glycoprotein that plays a crucial role in wound healing (Furie and Furie, 2005; Patten and Wang, 2020). It is a dimer consisting of two nearly identical monomers linked covalently at their C-termini by a pair of disulfide bonds. Each monomer has an approximate molecular weight of ∼250 kDa, consisting of a linear arrangement of three types (types I, II, and III) of repeating units (Erickson, 2002). Plasma fibronectin is a substrate of thrombin-activated coagulation factor XIII (FXIIIa, plasma transglutaminase), which can also be crosslinked to fibrin leading to structural alterations of the fibrin network (Jara et al., 2020). In the clot retraction process, fibronectin may interact with platelet α_IIb_β_3_, thereby regulating the interaction between platelets and fibrin (Huang et al., 2019). Adhesion of circulating platelets to matrix proteins including fibronectin and to fibronectin–fibrin clot can activate platelets, leading to the formation of more platelet thrombi and enhancement of platelet cohesion (Furie and Furie, 2005).

The influence of glycosylation on fibronectin has attracted continuous attention among immunologists (Sano et al., 2008; Freire-De-Lima et al., 2011; Park et al., 2011; Hsiao et al., 2017). It is reported that glycosylation can significantly affect the ligand recognition of fibronectin, suggesting a modulation role of glycans (Sano et al., 2008). O-GalNAc glycosylation of fibronectin is found to be associated with the epithelial–mesenchymal transition (EMT) process, where an O-glycan at a specific Thr of fibronectin (inside the type III homolog connective segment) induces the reactivity of monoclonal antibody (mAb) FDC6 (Freire-De-Lima et al., 2011). In addition, it was reported that O-GalNAc glycosylation may interfere with the intracellular degradation process of fibronectin after endocytosis, thus stabilizing fibronectin (Park et al., 2011). The study further suggested that the fibronectin O-glycosylation pathway may be an important factor in breast cancer development and progression (Park et al., 2011). Furthermore, another study indicated that poly-*N*-actetyllactosamine–containing glycans on fibronectin could decrease its binding affinity to gelatin (Sauerzapfe et al., 2009). A comprehensive glycosylation mapping of fibronectin can facilitate our understanding of the molecular details of these functions.

The initial glycoproteomics study of fibronectin was conducted 16 years ago by MALDI-TOF (Tajiri et al., 2005) glycopeptide analysis which was limited by conventional enrichment and MS fragmentation techniques. Recent advances in chromatography and mass spectrometry have greatly improved the efficiency of glycopeptide enrichment enabling high-resolution glycan analysis. Particularly, zwitterionic-hydrophilic interaction chromatography (ZIC-HILIC) has been applied extensively to improve glycopeptide enrichment with TFA added to the ACN/H_2_O mobile phase system as an ion-pairing reagent (Wohlgemuth et al., 2009; Alagesan et al., 2017). Orbitrap brings higher collision dissociation HCD, thus enabling high resolution and more fragmentation in tandem MS, which is now routinely used in modern peptide analysis (Michalski et al., 2012). In addition, stepped normalized collision energy (sNCE)-HCD was recently developed to further enhance glycopeptide fragmentation (Liu et al., 2017). Moreover, an O-glycan–specific protease-assisted method named “site-specific extraction of O-linked glycopeptides” (EXoO) was recently developed, which allows for the comprehensive analysis of O-glycosites and O-glycans (Yang et al., 2020). We have been using these advanced glycoproteomic techniques to elaborate glycosylation of key proteins in thrombosis, achieving site-specific glycan mapping of human factor FVIII (Qu et al., 2020) and factor FV (Ma et al., 2020).

In this study, we performed systematic glycan analysis of human plasma fibronectin by optimizing and applying an integrated approach we recently developed (Ma et al., 2020). Briefly, Glu-C and trypsin were used for peptide mapping, N-glycopeptides were enriched by ZIC-HILIC, and O-glycopeptides were enriched through EXoO (Yang et al., 2020). N-glycosites and O-glycosites were localized by ^18^O-labeling and EXoO (with or without sialidase), respectively. Optimized sNCE-HCD fragmentation (Zhu et al., 2020) was utilized to annotate *N*- and *O*-glycopeptide sequences. As a result, 308 unique glycopeptides comprising 6 N-glycosites and 53 O-glycosites were identified with simultaneous determination of peptide sequences and glycoform compositions.

## Materials and Methods

### Materials

Reagents, solvents, and chemicals were all from Sigma-Aldrich (St. Louis, MO) unless otherwise stated. Human plasma fibronectin with a purity of a minimum 90% was purchased from Haematologic Technologies (Essex Junction, VT). Trypsin (sequencing grade modified) and endoproteinase Glu-C (Glu-C) were from Promega (Madison, WI). PNGase F was obtained from New England Biolabs (Beverly, MA). The 10-kDa Microcon centrifugal filter devices were purchased from Millipore (Bedford, MA). The ZIC-HILIC material was from SeQuant (Umea, Sweden). The OpeRATOR/SialEXO kit was from Genovis, Inc. (Cambridge, MA).

### Sample Preparation and Enzymatic Digestion

Filter-aided sample preparation (FASP) was used to prepare samples for MS analysis. Briefly, the fibronectin protein was dissolved in 0.4% SDS, 50 mM dithiothreitol (DTT) in 50 mM NH_4_HCO_3_ (pH 7.8), which was incubated at 95°C for 10 min. The resulting solution was diluted by 200 μL of 8 M urea in 100 mM Tris-HCl buffer, pH 8.5 (UA solution), and then transferred to a 10-kDa ultracentrifuge filter for centrifugation at 13,500 g for 20 min. The concentrate was then mixed with another 200 μL of the UA solution and centrifuged twice. Subsequently, 50 μL of 50 mM iodoacetamide (IAA) in the UA solution was added, and the mixture was incubated in darkness at room temperature for 30 min, followed by brief centrifugation for 30 min. Then, the concentrate was diluted with 200 μL of UA solution and centrifuged twice to remove excess amounts of IAA. Finally, the sample was diluted with 100 μL of 40 mM NH_4_HCO_3_ and concentrated twice. After concentrating, the sample was digested with 1:50 trypsin:sample (w/w) in 40 mM NH_4_HCO_3_ (pH 7.8) overnight at 37°C. In protein ID identification, Glu-C was added at a 1:10 ratio (w/w) in the same buffer and incubated for 4 h at 37°C before trypsin digestion. Fibronectin peptides were eluted with 50 μL NH_4_HCO_3_ (pH 7.8) by centrifuging the filter units for 20 min. This step was repeated three times. The final concentration of peptides was determined by a UV spectrophotometer (Nanodrop, Thermo) using an extinction coefficient of 1.1 for 0.1% (g/L) solution at 280 nm.

### N-Glycopeptide Enrichment

N-glycopeptides were enriched by an in-house packed ZIC-HILIC micro-tip as previously described (Ma et al., 2020). The HILIC micro-tip was washed with 500 μL of ACN, water, and binding buffer (80% ACN/1% TFA) twice sequentially. For sample loading, the dried fibronectin peptides were dissolved in 500 μL of binding buffer and loaded onto the tip three times. The tip was then washed with 1.5 ml of binding buffer, and glycopeptides were eluted with 1 ml of elution buffer (0.1% TFA). The eluted solution was lyophilized and stored at -20°C until use. Enriched glycopeptides were directly injected into an LTQ-Orbitrap Elite mass spectrometer (MS) for intact glycopeptide analysis. For N-glycosite analysis, HILIC-enriched N-glycopeptides were incubated with PNGase F in 20 μL of 50 mM NH_4_HCO_3_ (pH 7.5) in H_2_^18^O at 37°C overnight. Deglycosylated peptides were desalted, dried, and stored at -20°C for mass spectrometry analysis.

### O-Glycopeptide Analysis

The EXoO method was used to identify O-glycoforms and O-glycosites as previously reported (Yang et al., 2020). Briefly, 200 μL of AminoLink resin (Pierce, Rockford, IL) was incubated with 500 μg of digested peptides in 50 mM NaCNBH_3_ and 50 mM NaH_2_PO_4_ (pH 7.5) overnight at room temperature. The resin was then washed in a spin column and blocked by 1 M Tris-HCl (pH 7.4) and 50 mM NaCNBH_3_ at room temperature for 30 min. After washing, the O-glycopeptides were released from the resin by the O-glycan–specific protease OpeRATOR (1 unit per 1 μg peptides) in 20 mM Tris-HCl (pH 6.8) at 37°C for 15 h. The released O-glycopeptides were desalted and dried for MS analysis. For O-glycosite analysis, sialidase was added together with OpeRATOR, so O-glycoforms would be unified and thus enhance the signal of O-glycopeptides.

### LC−MS/MS Analysis of Intact Glycopeptides

Experiments were performed on an LTQ-Orbitrap Elite MS equipped with an EASY-Spray source and a nano-LC UltiMate 3000 high-performance liquid chromatography system (Thermo Fisher). An EASY-Spray PepMap C18 column (length, 15 cm; particle size, 3 μm; pore size, 100 Å; Thermo Fisher) was used for separation. The separation was achieved under a linear gradient elution condition from 3 to 40% solvent B for 30 min at a flow rate of 300 nL/min (mobile phase A, 2% ACN, 98% H_2_O, and 0.1% FA; mobile phase B, 80% ACN, 20% H_2_O, and 0.1% FA). LTQ-Orbitrap Elite was operated in the data-dependent mode, and the ten most intense ions in MS^1^ were subjected to HCD 30 in the HCD collision cell for deglycosylated peptide analysis, or stepped normalized collision energy (sNCE)-HCD 15–30–45 fragmentation for intact glycopeptide analysis. The Orbitrap MS acquired a full-scan survey (m/z range from 375 to 1,500; automatic gain control target, 10^6^ ions; resolution of 60,000 at m/z 400; maximum ion accumulation time, 50 ms). For sNCE-HCD, the Orbitrap analyzer acquired HCD fragment ion spectra with a resolution of 15,000 at m/z 400 (automatic gain control target, 10,000 ions; maximum ion accumulation time, 200 ms). The MS/MS scan model was set as the centroid. Other conditions used include an S-lens RF level of ∼60% and an ion selection threshold of 50,000 counts for HCD.

### Data Analysis

Data analysis of *N*-glycosite mapping was performed by pFind 3.0 (Chi et al., 2018). Peptide fragments were matched against the fibronectin protein sequence (UniProtKB entry P02751), where iodoacetamide on Cys was set as a static modification and oxidation of Met and ^18^O-labeling of Asn (m = 2.9848) were set as a dynamic modification. Trypsin and Glu-C were chosen as the enzyme, and two missed cleavages were allowed. A false discovery rate (FDR) of 1% was estimated and applied at the peptide level. pGlyco 2.0 was applied for intact *N*-glycopeptide analysis (Liu et al., 2017). O-glycosylation results were manually interpreted with the assistance of GPQuest (Toghi Eshghi et al., 2015). Briefly, the MS/MS spectra containing at least two of the oxonium ions of HexNAc, including m/z = 126.05, 138.05, 144.06, 168.06, 186.08, and 204.08, were selected as glycopeptide spectra. The presence of at least 30% of b or y ions and three intact glycopeptide ions was required (Toghi Eshghi et al., 2015). The theoretical peptide database was constructed by using Lys/Arg on the C-terminal side (trypsin digestion) followed by Ser/Thr (OpeRATOR digestion) on the N-terminal side with four miss-cleavage sites allowed. In all analysis, the mass tolerance was set at 10 ppm for precursor ions and 50 ppm for product ions. Relative quantitative analysis was performed by comparing the peak area of ion chromatograms extracted from Xcalibur. The mass spectrometry data have been deposited to the ProteomeXchange Consortium *via* the PRIDE partner repository (Vizcaíno et al., 2014) with the dataset identifier PXD025886.

## Results and Discussion

### Protein and Glycopeptide Identification

**FIGURE 1:**
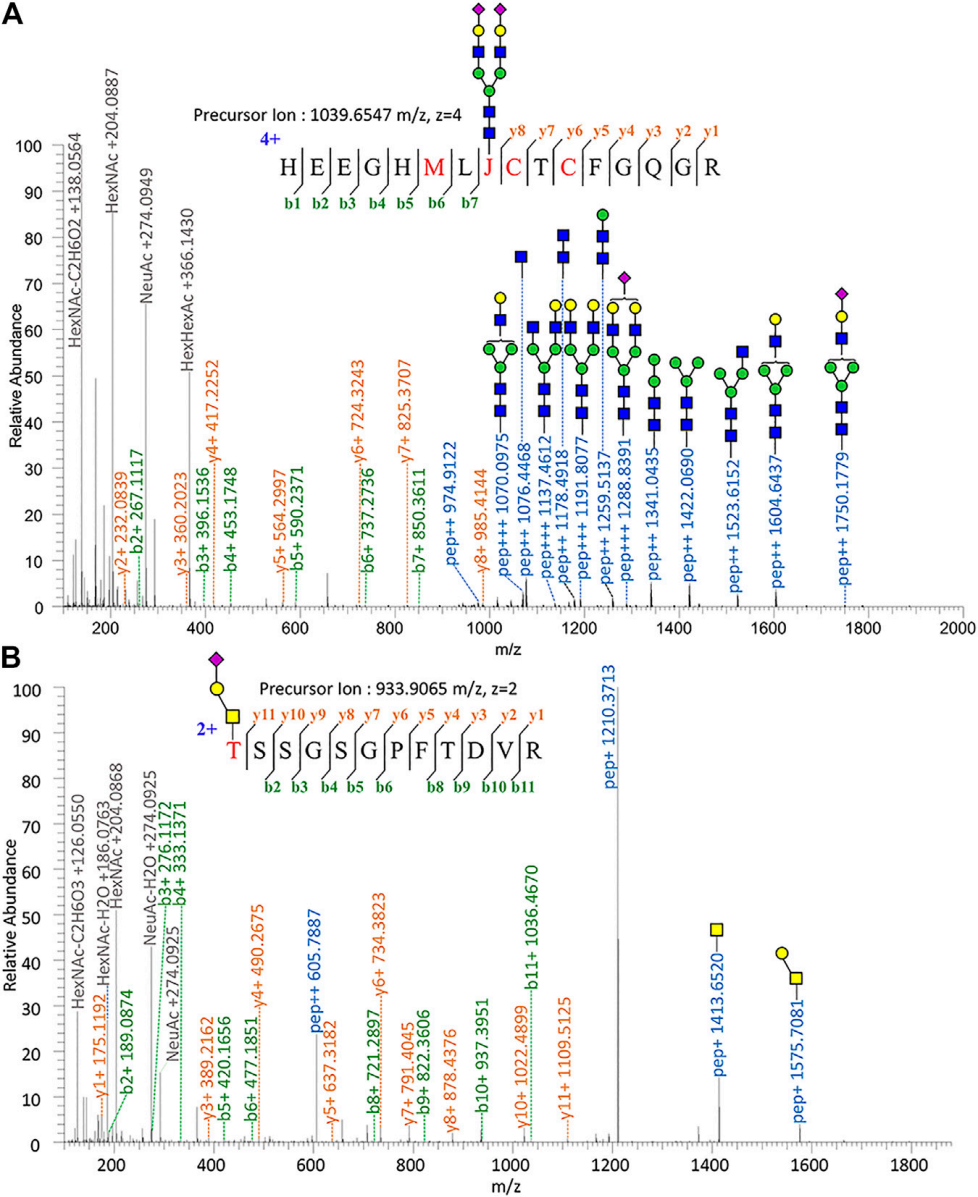
Tandem mass spectrum annotation of fibronectin glycopeptides. **(A)** N-glycopeptide HEEGHMLJ^542^CTCFGQG R and **(B)** O-glycopeptide T^279^SSGSGPFTDVR.

Proteomic analysis of Glu-C-tryptic–digested fibronectin confirmed the identity of plasma fibronectin. The sequence coverage of the full-length glycoprotein (fibronectin, 2475 AAs, ∼500 kDa) is 90.35% with minimal protein impurities. ZIC-HILIC enabled the enrichment of most *N*-glycopeptides, and sNCE-HCD fragmentations enabled the determination of both peptide sequences and glycoform compositions in one spectrum. From our previous optimization, HCD 15–30–45 was the most suitable condition for Orbitrap Elite (Zhu et al., 2020). For example, the N-glycopeptide HEEGHMLJ*CTCFGQGR (Asn542) of fibronectin with disialylated complex type N-glycan was fully annotated from the fragment information (Figure 1A) and Asn with glycosylation was replaced by “J” in data analysis. Oxonium ions of HexNAc (m/z = 204.08) could be detected under our fragmentation method, including m/z = 186.08, 144.07, 138.05, and 126.05 (Figure 1) (Halim et al., 2014). The oxonium ion of Neu5Ac (m/z = 274.09 or 292.10) was used to determine the presence of sialic acid. Diagnostic ions of HexHexNAcFuc (m/z = 512.19 Da) were applied to define the fucose branch (Supplementary Figure S3). A core-fucose structure was determined by the PepHexNAcFuc (+) ion and its neutral loss of fucose ion (146.06 Da) PepHexNAc (+) (Supplementary Figure S1) (Zhou et al., 2017).

Since OpeRATOR digests O-glycopeptides at the N-terminus of O-glycosylated Ser or Thr, the EXoO method enables the enrichment of O-glycopeptides as well as the identification of O-glycan localization (O-glycosites). Similarly, sNCE-HCD was used to profile fibronectin O-glycopeptides, as shown in Figure 1B showing the spectrum of O-glycopeptide T*SSGSGPFTDVR (Thr279). Tandem MS annotations of other O-glycopeptides are provided in Supplementary Figures S14–S21.

### N-Glycosylation Profiling and Relative Quantification

**FIGURE 2:**
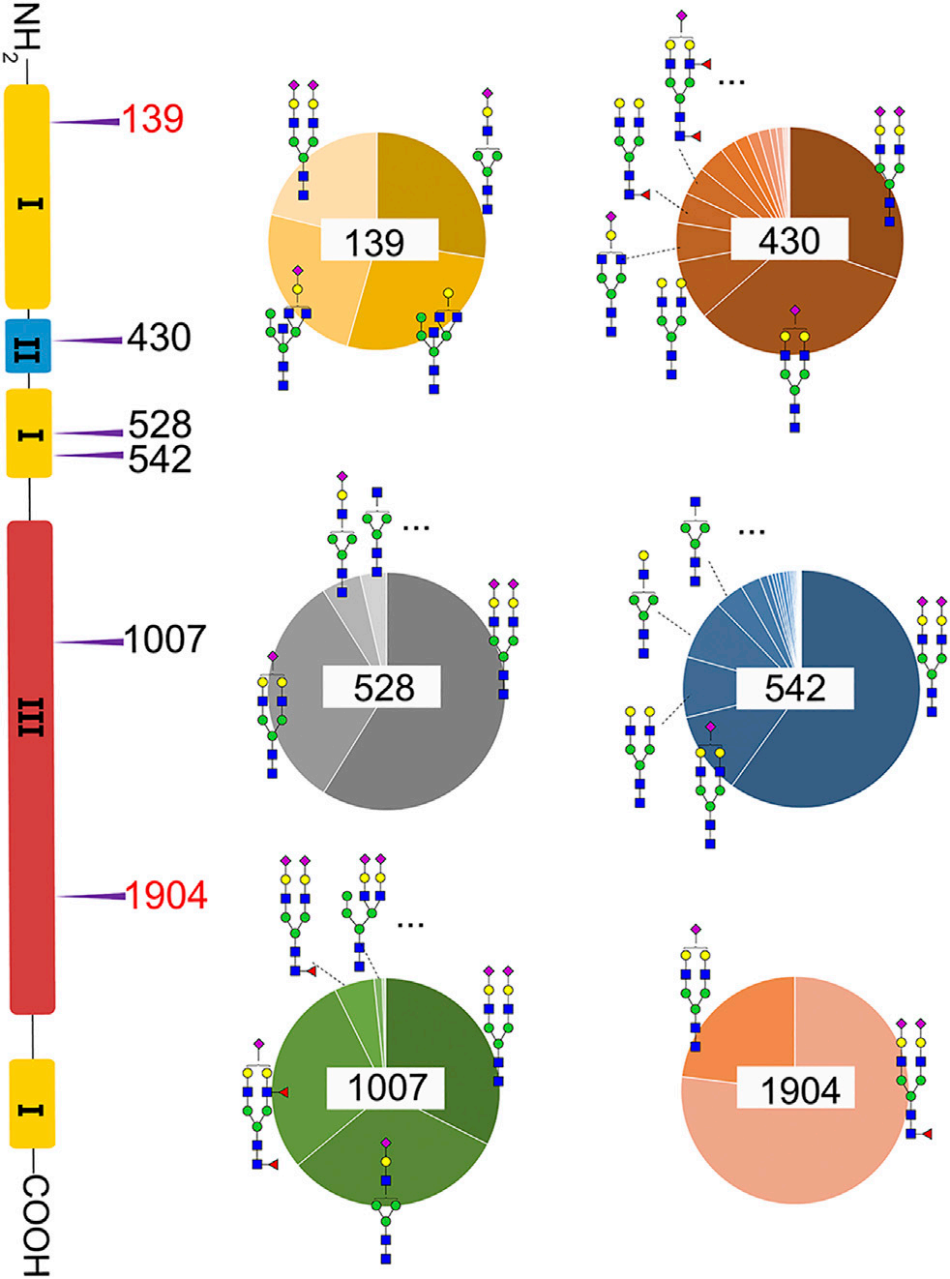
Microheterogeneity and relative abundance of *N*-glycoforms at six fibronectin *N*-glycosites. Only two glycoforms were identified at Asn1904. Two N-glycosites (red color) were newly identified sites. 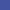: GlcNAc; 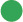: Man; 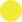: Gal; 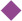: Neu5Ac; 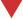: Fuc.

A total of 82 unique *N*-glycopeptides were identified from 302 MS spectra of HILIC-enriched samples, which contain 38 site-specific N-glycoforms on 6 *N*-glycosites as evidenced by HCD fragmentation (Figure 2, Supplementary Table S1). Three *N*-glycosites were identified on type I domains, two *N*-glycosites were identified on type III domains, and only one *N*-glycosite was found on type II domains. In addition, four or more glycoforms were identified on five *N*-glycosites, suggesting a glycan microheterogeneity of fibronectin. Among the six *N*-glycosites, Asn542 located within type I domain 8 was the most heterogeneous site with 27 different N-glycoforms. Sialyated glycans are predominant on this site. The second most heterogenous N-glycosite is Asn430 within type II domain 2, which presents 17 different glycoforms. Additionally, eight, four, and four glycoforms were identified on N-glycosites Asn1007, Asn528, and Asn139, respectively. Interestingly, Asn1904 contained only two glycoforms with structural similarity (N4H5S1, N4H5F1S2).

**FIGURE 3:**
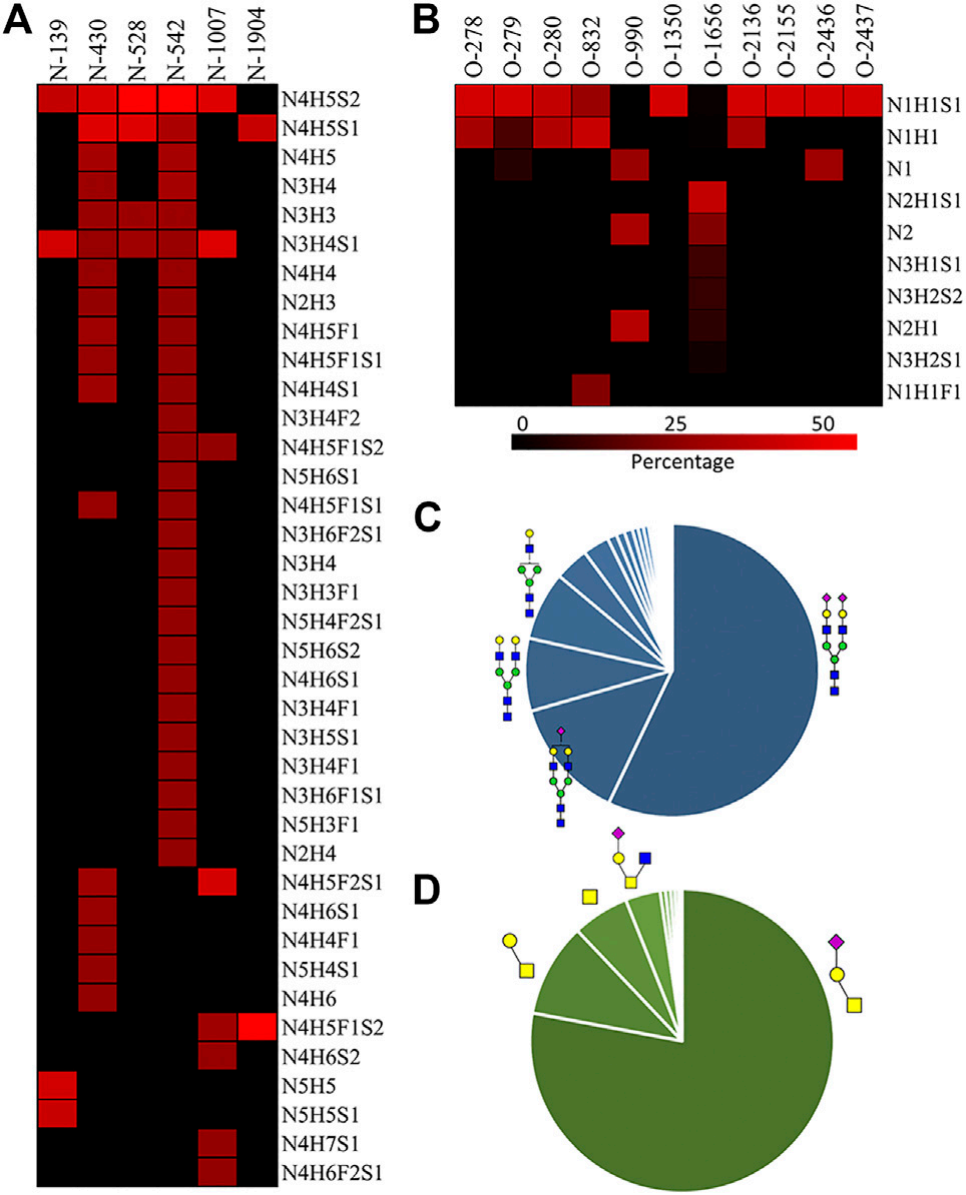
**A)** N-glycan percentage of each N-glycosite; **(B)** O-glycan percentage of each O-glycosite; **(C)** overall N-glycan percentage; **(D)** overall O-glycan percentage. 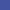: GlcNAc; 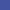: GalNAc; 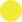: Gal; 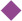: Neu5Ac; 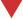: Fuc.

We then quantified the overall relative abundance of all *N*-glycoforms as well as the relative abundance of *N*-glycoforms at each *N*-glycosite. Ion chromatograms of identified peptides extracted from Xcalibur were used to quantify different glycopeptides. The mass-to-charge ratio (m/z) and retention time (RT) of glycopeptide precursor ions identified above were used to extract ion chromatograms and calculate the peak area of individual glycopeptides. Peak areas were then normalized to obtain site-specific relative abundances of each *N*-glycoform as shown in pie charts (Figure 2). Among 27 *N*-glycoforms identified at Asn542, disialylated bi-antennary N4H5S2 (**21**) and monosialylated bi-antennary N4H5S1 (**20**) are highly abundant, with 59.93 and 11.23%, respectively. The top three abundant *N*-glycans on site Asn430 are similar to those on Asn542 with different abundance, including N4H5S1 (**20**, 33.11%), N4H5S2 (**21**, 30.42%), and N4H5 (**13**, 8.48%). Asn1007 also contains a high percentage of N4H5S2 (**21**, 32.51%) but also other glycoforms such as N3H4S1 (**10**, 31.47%) and N4H5F2S1 (**19**, 28.70%). Figures 3A and C illustrate the overall distribution of fibronectin *N*-glycans. The top two abundant *N*-glycans are all complex types, including N4H5S2 (**21**, 57.09%) found on most sites and N4H5S1 (**20**, 13.43%) identified on Asn430, Asn528, Asn542, and Asn1904.

Another observation is that most multi-antennary *N*-glycans are in relatively low abundance. The most abundant tri-antennary hybrid type *N*-glycan N5H5 (**23**, 26.87%) and its monosialylated form N5H5S1 (**27**, 24.52%) are all attached on Asn139, and both carry the bisecting GlcNAc. It was reported that fibronectin binds to transmembrane receptor protein integrins (Pankov and Yamada, 2002), and the existence of the bisecting GlcNAc on the α5 subunit could considerably diminish the adhesion of integrin α5β1 to fibronectin (Takahashi et al., 2009). It is thus reasonable to speculate that Asn139 within type I domain 3 and the glycans on this site could play a critical role in the integrin–fibronectin interaction, as no bisecting glycans were identified on other glycosites.

We observed that fibronectin is highly sialylated, with 77.31% of identified N-glycans containing one (19.68%) or two (57.63%) sialic acid residues. Sialic acid can affect conformation and oligomerization and the interaction function of proteins (Bhide and Colley, 2017). The existence of sialic acid on proteins affects their absorption, half-life, and clearance from the serum, as well as the physical, chemical, and immunogenic properties (Bork et al., 2009). Besides, high degrees of sialylation were found to play functional roles in heamostasis glycoproteins. For example, sialylation of the α5β1 integrin can decrease its binding affinity to fibronectin, thereby affecting cell adhesion in myeloid cells (Pan and Song, 2010). In addition, another hemostasis protein von Willebrand factor (VWF) was reported to be highly sialylated, which can interact with the asialoglycoprotein receptor (ASGPR) in the liver (O'Sullivan et al., 2016). It is possible that high sialylation of fibronectin might affect its interaction with certain extracellular matrix proteins, including collagen (Teoh et al., 2018), fibrin (Makogonenko et al., 2002), or fibulin-1 (Gu et al., 2000).

Compared with previously reported N-glycosylations of fibronectin (Tajiri et al., 2005), two new sites were discovered, including Asn139 and Asn1904. Surprisingly, Asn139 was found to be located in the Asn-Xxx-Cys sequence instead of the consensus site Asn-Xxx-Ser/Thr. The N-glycosite in Asn-Xxx-Cys sequences was not noticed until recently. This atypical N-glycosite was discovered in human protein C and human GPR109A, which significantly affected the protein functions (Gil et al., 2009; Yasuda et al., 2015). It was noticed that three previously identified sites were not observed in intact glycopeptide analysis, including Asn877, Asn1244, and Asn2199. These sites are located in theoretical tryptic-digested long peptide chains, which were hardly observed using the current intact glycopeptide analysis method. Thereby, Glu-C was used to further shorten the peptide chain and determine the sites through ^18^O-labeling. The peptide fragmentation results of the three sites with ^18^O-labeled glycosylation sites are shown in Supplementary Figures S11–S13. Unfortunately, we were not able to identify intact glycopeptides after Glu-C and trypsin combined digestion due to software limitations. The peptide fragmentation results with other ^18^O-labeled glycosylation sites are shown in Supplementary Figures S5–S10.

### O-Glycosylation Profiling and Relative Quantification

**FIGURE 4:**
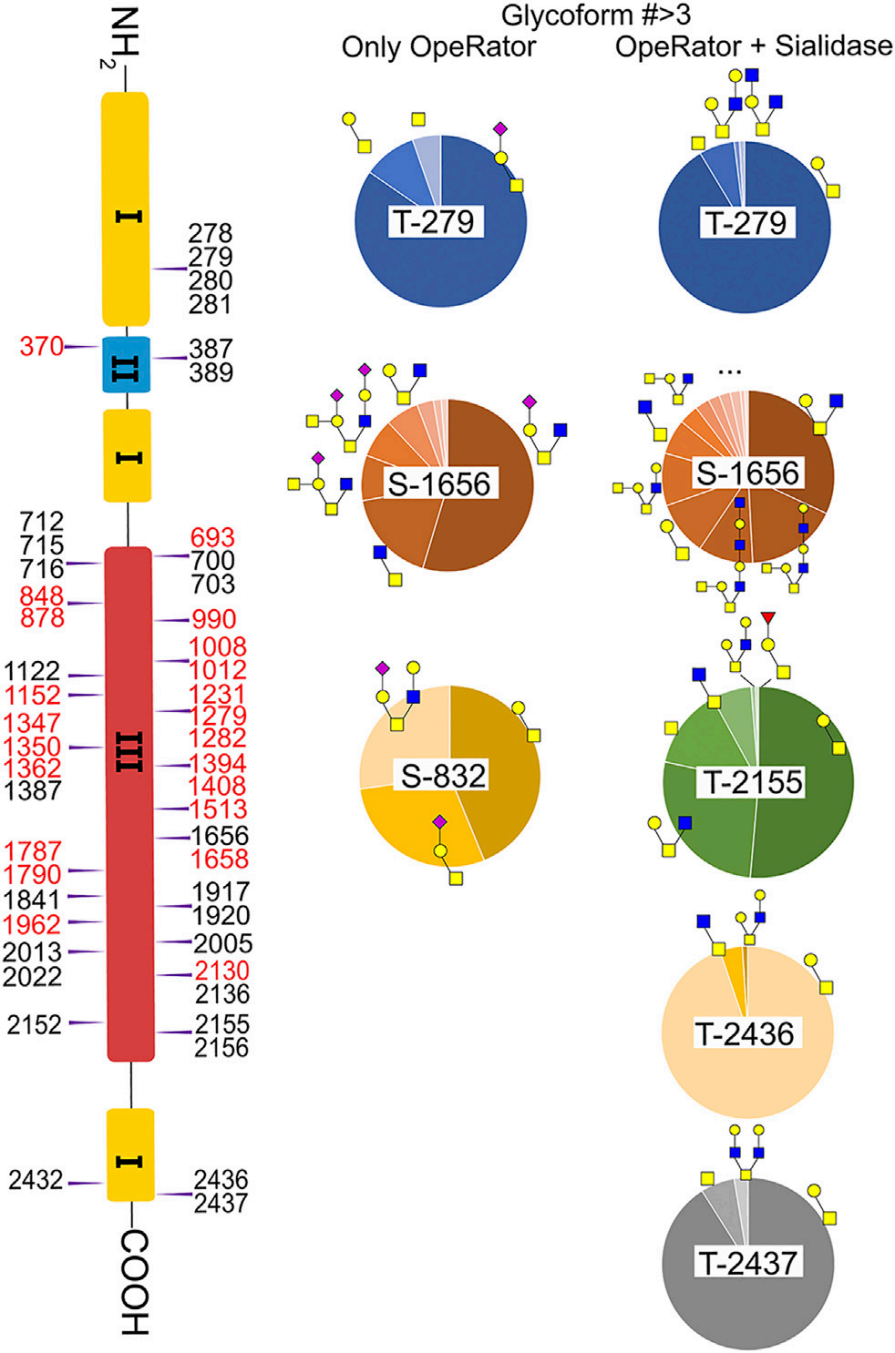
Identified *O*-glycosites and glycoforms on each site in fibronectin. Red highlighted glycosites are new sites localized in this report. 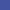: GlcNAc; 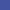: GalNAc; 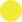: Gal; 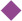: Neu5Ac; 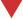: Fuc.

Fibronectin was reported as one of the most highly *O*-glycosylated proteins in the blood, with 71 possible sites (lacking glycoform information) identified from human serum samples. However, only 28 of those sites were precisely localized *via* a lectin enrichment method (King et al., 2017). Here, we localized the exact O-glycosites by using the EXoO method (Ma et al., 2020). O-glycoforms at each site were also identified and relatively quantified by using the modified EXoO method. In total, 16 different O-glycoforms on 53 O-glycosites were identified on fibronectin. Among the 53 O-glycosites, 11 were previously localized and 14 were identified before without exact localization information (King et al., 2017). The other 28 O-glycosites were never identified before. Specifically, our method enabled the identification of a new O-glycosite on type I domains (Ser281), a new site on type II domains (Thr370), and 26 sites on type III domains (Figure 4). Collectively, 43 identified O-glycosites are located within type III domains and 10 O-glycosites were distributed on other domains. We did not detect the other 17 possible O-glycosites, which may be due to different glycopeptide enrichment methods (Ma et al., 2020). The core 1 and Tn structures (Supplementary Table S2, structures **39**–42) were the major O-glycoforms observed on most sites, but core 2, core 3, and core 4 *O*-glycans were also identified (Supplementary Tables S2, S3, **43**–**59**).

In this study, the EXoO method with or without sialidase was used to study the O-glycosylation of fibronectin (Yang et al., 2020). Sialic acid information of the O-glycoforms was obtained without sialidase treatment, but treatment with sialidase enabled the identification of more O-glycosites and glycoforms. Anyway, we successfully identified 10 O-glycoforms (5 are sialylated) on 11 O-glycosites using the EXoO methods without sialidase treatment (Supplementary Table S2). This is the first example of determining complete O-glycan structures with sialic acid information on glycoproteins, as the previously reported EXoO method requires de-sialylation (Ma et al., 2020). Additional sialidase treatment enabled identifying 6 more glycoforms and 43 more O-glycosites (Supplementary Table S3). For example, as shown in Figure 4, Thr279 was found mainly occupied by the sialyl-T antigen with also T and Tn antigens identified when using the non-sialidase–treated method, while some low abundant core 2 structures were also observed at the site when using the sialidase-treated method. Most strikingly, Thr2155 was only detected to have sialyl-T antigen without sialidase treatment but was found to be the second most complex O-glycosite with six glycoforms using sialidase-treated EXoO (Figure 4). Interestingly, the poly-LacNAc structure that could enhance the binding affinity between gelatin and fibronectin (Sauerzapfe et al., 2009) was possibly identified in core 2 O-glycans on Ser1656. However, the ExoO method cannot exclude multiple O-glycosites for peptides with repetitive sequences (Yang et al., 2020).

The relative abundance of glycoforms at each O-glycosite was also determined through peak area integration using the same methods for determining N-glycoform abundance. The relative abundance of O-glycans on some sites (>3 O-glycoforms) is shown in pie charts in Figure 4. Among O-glycoforms identified at Thr279, sialyl-T antigen N1H1S1 (**41**), T antigen N1H1 (**40**), and Tn antigen N1 (**39**) occupy 84.6, 10.1, and 5.3% of total glycoforms, respectively. The most heterogeneous O-glycosite Ser1656 contains sialylated core 2 structure N2H1S1 (**43**, 54.6%), asialyated core 3 structure N2 (**45**, 17.6%), sialylated core 4 structure N3H1S1 (**48**, 8.5%), disialylated core 4 structure N3H2S2 (**46**, 7.3%), core 2 structure N2H1 (**44**, 6.3%), and 13 other glycoforms. Figure 3D shows the overall distribution of fibronectin O-glycans identified by the non-sialidase–treated EXoO method. The top two abundant glycoforms are both core 1 structures. The most abundant structure is sialyl-T antigen (78.0%), which was identified on 10 O-glycosites. The second abundant glycoform is the T antigen (10.0%) that distributed on six *O*-glycosites. The Tn antigen (46.0%) is the third abundant O-glycoform, which was found on three O-glycosites.

O-glycosylation of fibronectin was proven to determine its binding to mAb FDC-6 (Nichols et al., 1986). Moreover, a recent study found that the key O-glycosylation is located in the type III homology connective segment (IIICS) domain (Freire-De-Lima et al., 2011). Furthermore, O-glycanase–treated fibronectin showed a reduced affinity with FDC-6, suggesting that this binding might associate with less complex asialylated O-glycans (Feinberg and Wang, 1994). However, our data showed that the previously identified site T2155 is primarily occupied by the sialyl-T antigen (Figure 3B) and thus may not associate with the binding to FDC-6. Here, we identified three other O-glycosites in the type IIICS domain, including T2130, S2136, and T2152, all occupied with asialylated glycoforms, which may thus associate with the binding to FDC-6.

Sialylation on O-glycans was found to play key roles in molecular interactions and protein functions. For example, platelets clearance is necessary for normal hemostasis in humans, and reduced sialylation of O-glycans in platelets causes increased clearance in the liver (Li et al., 2017). The sialylation of O-glycans on cell surface CD8 negatively affects the binding affinity between histocompatibility complex class I molecules (MHCI) and CD8 (Moody et al., 2001). Similar to N-glycans, O-glycans of fibronectin were also highly sialylated. As shown in Figure 3D, 82.88% of total O-glycans were sialylated. Such a high O-glycan sialylation could possibly influence the function of fibronectin or interactions with other biomolecules.

## Conclusion

In summary, we performed site-specific N- and O-glycosylation analyses of human plasma fibronectin through an integrated strategy. Multi-enzyme digestion, ZIC-HILIC enrichment, and sNCE-HCD fragmentation enabled complete annotation of fibronectin glycopeptides. In total, 82 unique N-glycopeptides and 226 O-glycopeptides were detected from enriched fibronectin samples. From the glycopeptides, we identified 6 N-glycosites carrying 38 N-glycoforms and 53 O-glycosites carrying 16 O-glycoforms. The glycosite includes 2 new N-glycosites (one is an atypical Asn-Xxx-Cys site) and 28 new O-glycosites. Furthermore, complete O-glycan structures with sialic acid information were identified for the first time. The comprehensive N- and O-glycosylation mapping fills a knowledge gap of fibronectin and could facilitate its functional studies as well as fibronectin-related therapeutics development.

## Data Availability

The original contributions presented in the study are included in the article/Supplementary Material, and further inquiries can be directed to the corresponding author.
