# Supplementary material for: Site-Specific N- and O-Glycosylation Analysis of Human Plasma Fibronectin
Source: Front Chem. 2021 Jun 15;9:691217. doi: 10.3389/fchem.2021.691217 (PMC8239226; doi:10.3389/fchem.2021.691217)
Supplement: Supplementary file 1 [file DataSheet1.docx]

**Supplementary Information**

Site-specific N- and O-glycosylation Analysis of Human Plasma Fibronectin

Ding Liu^1^, Shuaishuai Wang^1^, Junping Zhang,^2^ Weidong Xiao^2^, Carol H. Miao,^3^ Barbara A. Konkle,^4^ Xiu-Feng Wan,^5,6,7,8^ Lei Li^1^*

^1^Department of Chemistry, Georgia State University, Atlanta, GA

^2^School of medicine, Indiana University, Indianapolis, IN

^3^Center for Immunity and Immunotherapies, Seattle Children's Research Institute, Seattle, WA

^4^Bloodworks Northwest, Seattle, WA

^5^Center for Influenza and Emerging Infectious Diseases (CIEID), University of Missouri, Columbia, MO

^6^Department of Molecular Microbiology and Immunology, School of Medicine, University of Missouri, Columbia, MO

^7^Bond Life Sciences Center, University of Missouri, Columbia, MO

^8^Department of Electrical Engineering & Computer Science, College of Engineering, University of Missouri, Columbia, MO

Correspondence: [lli22@gsu.edu](mailto:lli22@gsu.edu) (L.L.).

Table of Contents

| Section | Pages |
| --- | --- |
| Table S1. List of identified N-glycoforms and N-glycosites from Fibronectin | S2 |
| Table S2. List of identified O-glycoforms and O-glycosites from Fibronectin without sialidase treatment | S3 |
| Table S3. List of identified O-glycoforms and O-glycosites from Fibronectin with sialidase treatment | S4 |
| Figure S1-4. Tandem Mass spectra annotation of N-glycopeptides | S5-6 |
| Figure S5-S13. Tandem Mass spectra annotation of 18O labeled N-glycosylation sites | S7-S9 |
| Figure S14-S21. Tandem Mass spectra annotation of O-glycopeptides | S10-S13 |

**Table S1.** List of 38 identified N-glycoforms and 6 N-glycosites from Fibronectin**.**
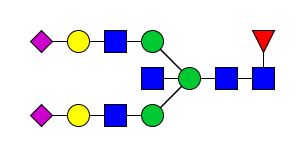
 N-acetylglucosamine (GlcNAc),
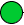
 Mannose (Man),
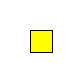
 N-acetylgalactosamine (GalNAc),
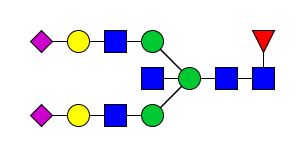
 Galactose (Gal),
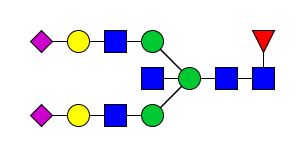
 N-acetylneuraminic acid (Neu5Ac),
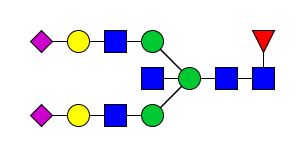
 L-Fucose

| **No.** | **Glycoform** | **Glycosite** |  | **No.** | **Glycoform** | **Glycosite** |  | **No.** | **Glycoform** | **Glycosite** |
| --- | --- | --- | --- | --- | --- | --- | --- | --- | --- | --- |
| **1** | **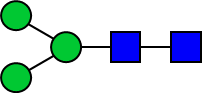** | 430, 542 |  | **15** | **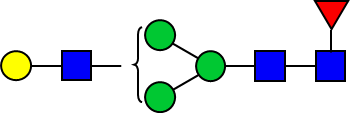** | 542 |  | **29** | **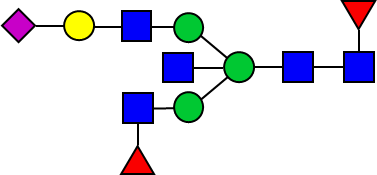** | 542 |
| **2** | **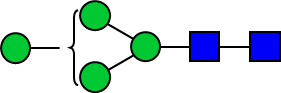** | 542 |  | **16** | **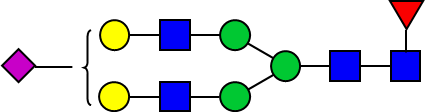** | 430, 542 |  | **30** | **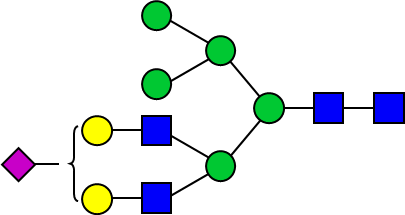** | 1007 |
| **3** | **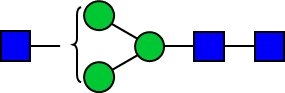** | 430, 528, 542 |  | **17** | **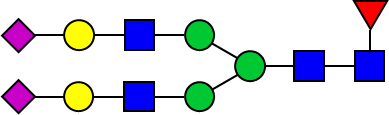** | 1007, 1904 |  | **31** | **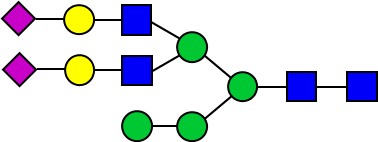** | 1007 |
| **4** | **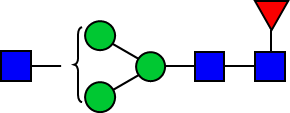** | 542 |  | **18** | **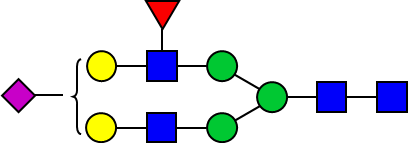** | 430, 542 |  | **32** | **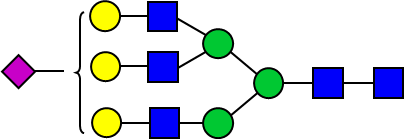** | 542 |
| **5** | **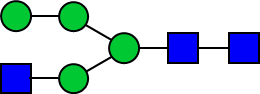** | 542 |  | **19** | **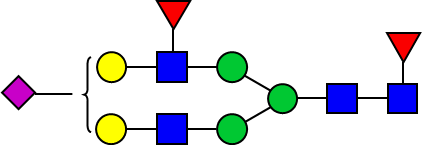** | 430, 1007 |  | **33** | **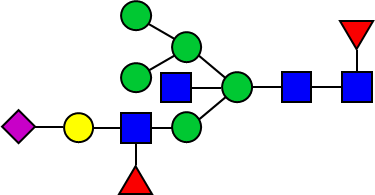** | 1007 |
| **6** | **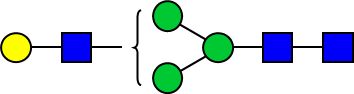** | 430, 542 |  | **20** | **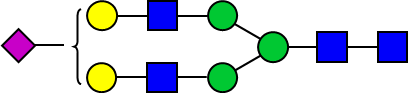** | 430, 528, 542, 1904 |  | **34** | 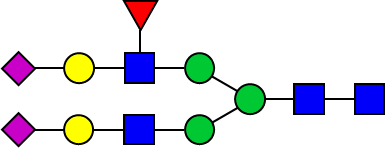 | 542, 1007 |
| **7** | **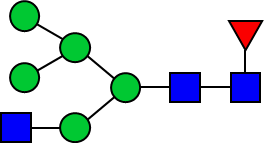** | 542 |  | **21** | **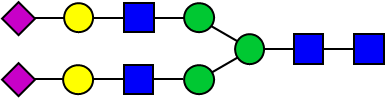** | 139, 430, 528, 542, 1007 |  | **35** | **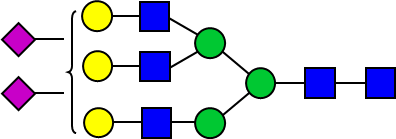** | 542 |
| **8** | **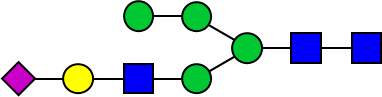** | 542 |  | **22** | **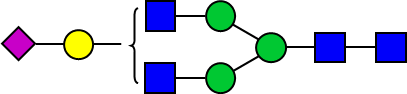** | 430, 542 |  | **36** | **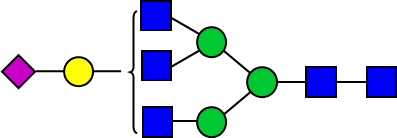** | 430 |
| **9** | **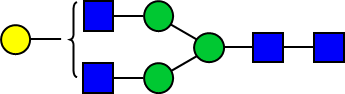** | 430, 542 |  | **23** | **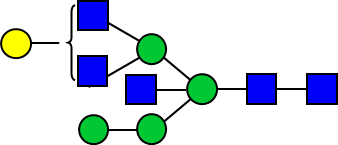** | 139 |  | **37** | **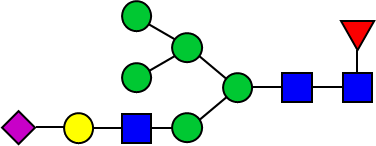** | 542 |
| **10** | **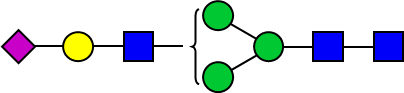** | 139, 430, 528, 542, 1007 |  | **24** | **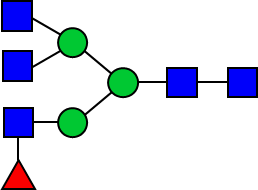** | 542 |  | **38** | **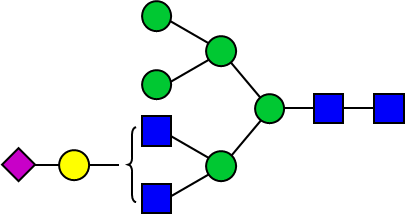** | 542 |
| **11** | **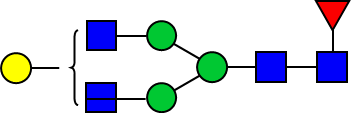** | 430 |  | **25** | **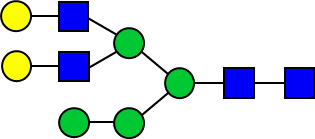** | 430 |  |  |  |  |
| **12** | **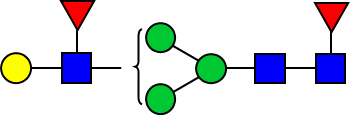** | 542 |  | **26** | **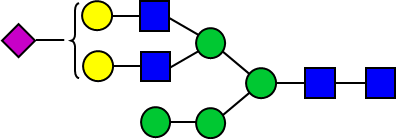** | 430 |  |  |  |  |
| **13** | **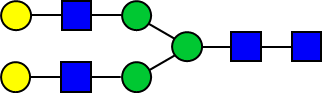** | 430, 542 |  | **27** | **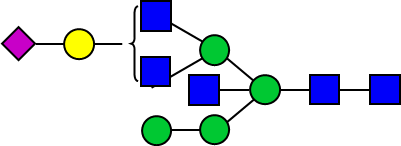** | 139 |  |  |  |  |
| **14** | **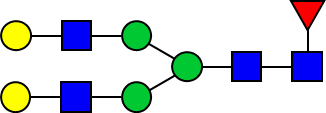** | 430, 542 |  | **28** | **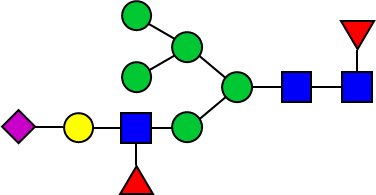** | 542 |  |  |  |  |

**Table S2**. List of 10 identified O-glycoforms and 11 O-glycosites from Fibronectin before sialidase treatment.
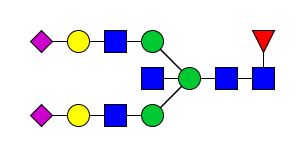
 N-acetylglucosamine (GlcNAc),
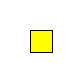
 N-acetylgalactosamine (GalNAc),
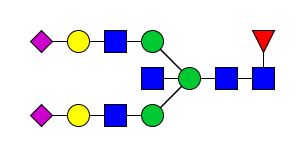
 Galactose (Gal),
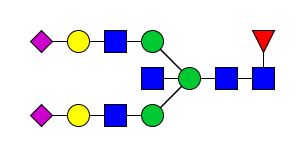
 N-acetylneuraminic acid (Neu5Ac),
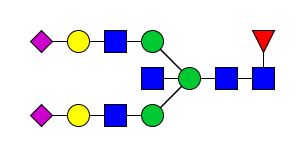
 L-Fucose (*multiple O-glycosites for peptides with repetitive sequences cannot be excluded)

| **No.** | **Glycoform** | **Glycosite** |  |  | **No.** | **Glycoform** | **Glycosite** |
| --- | --- | --- | --- | --- | --- | --- | --- |
| **39** | **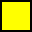** | T279, T990, T2436 |  |  | **46** | **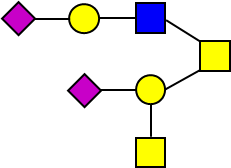** | *S1656 |
| **40** | **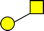** | T278, T279, S280, S832, S1656, S2136 |  |  | **47** | **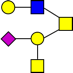** | *S1656 |
| **41** | **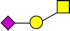** | T278, T279, S280, S832, T1350, S1656, S2136, T2155, T2436, T2437 |  |  | **48** | **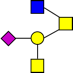** | *S1656 |
| **42** | **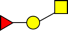** | S832 |  |  |  |  |  |
| **43** | **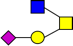** | *S1656 |  |  |  |  |  |
| **44** | **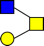** | T990, *S1656 |  |  |  |  |  |
| **45** | **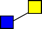** | T990, *S1656 |  |  |  |  |  |

**Table S3**. List of 16 identified O-glycoforms and 53 O-glycosites from Fibronectin after sialidase treatment.
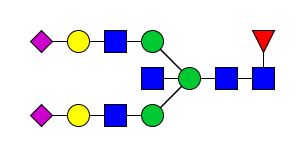
 N-acetylglucosamine (GlcNAc),
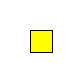
 N-acetylgalactosamine (GalNAc),
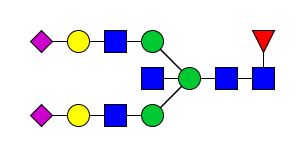
 Galactose (Gal),
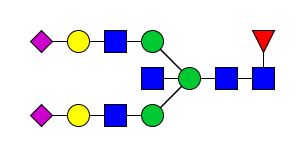
 N-acetylneuraminic acid (Neu5Ac),
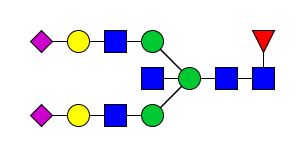
 L-Fucose (*multiple O-glycosites for peptides with repetitive sequences cannot be excluded)

| **No.** | **Glycoform** | **Glycosite** |  |  | **No.** | **Glycoform** | **Glycosite** |
| --- | --- | --- | --- | --- | --- | --- | --- |
| **39** | **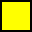** | T279, S280, S832, T2436, T990, S1122, T1513, T2130, S2136, T2155, T2437 |  |  | **54** | **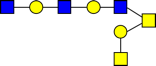** | S1656 |
| **40** | **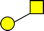** | T279, S280, S281, T370, S387, T389, T693, T700, S703, T712, T715, T716, T771, T773, T808, T818, S832, S848, T879, T990, T1009, T1012, S1122, T1152, S1231, S1347, T1350, T1362, T1387, S1394, S1408, T1513, S1656, S1658, S1787, T1790, T1841, T1917, T1920, T1962, S2013, T2022, T2130, S2136, T2152, T2155, S2432, T2436, T2437 |  |  | **55** | **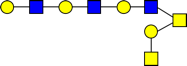** | *S1656 |
| **42** | **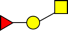** | T2155 |  |  | **56** | **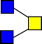** | *S1656 |
| **44** | **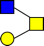** | T1279, S1282, *S1656, T2155 |  |  | **57** | **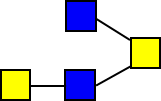** | *S1656 |
| **45** | **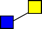** | *S1656, T2155, T2436 |  |  | **58** | **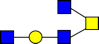** | *S1656 |
| **49** | **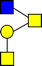** | T279, *S1656 |  |  | **59** | **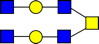** | *S1656 |
| **50** | **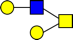** | T278, T279, T1012, *T2005, T2155, *T2436 |  |  |  |  |  |
| **51** | **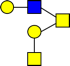** | T1279, *S1656, T2437 |  |  |  |  |  |
| **52** | **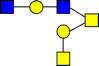** | *S1656 |  |  |  |  |  |
| **53** | **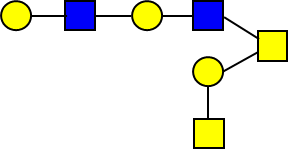** | *S1656 |  |  |  |  |  |


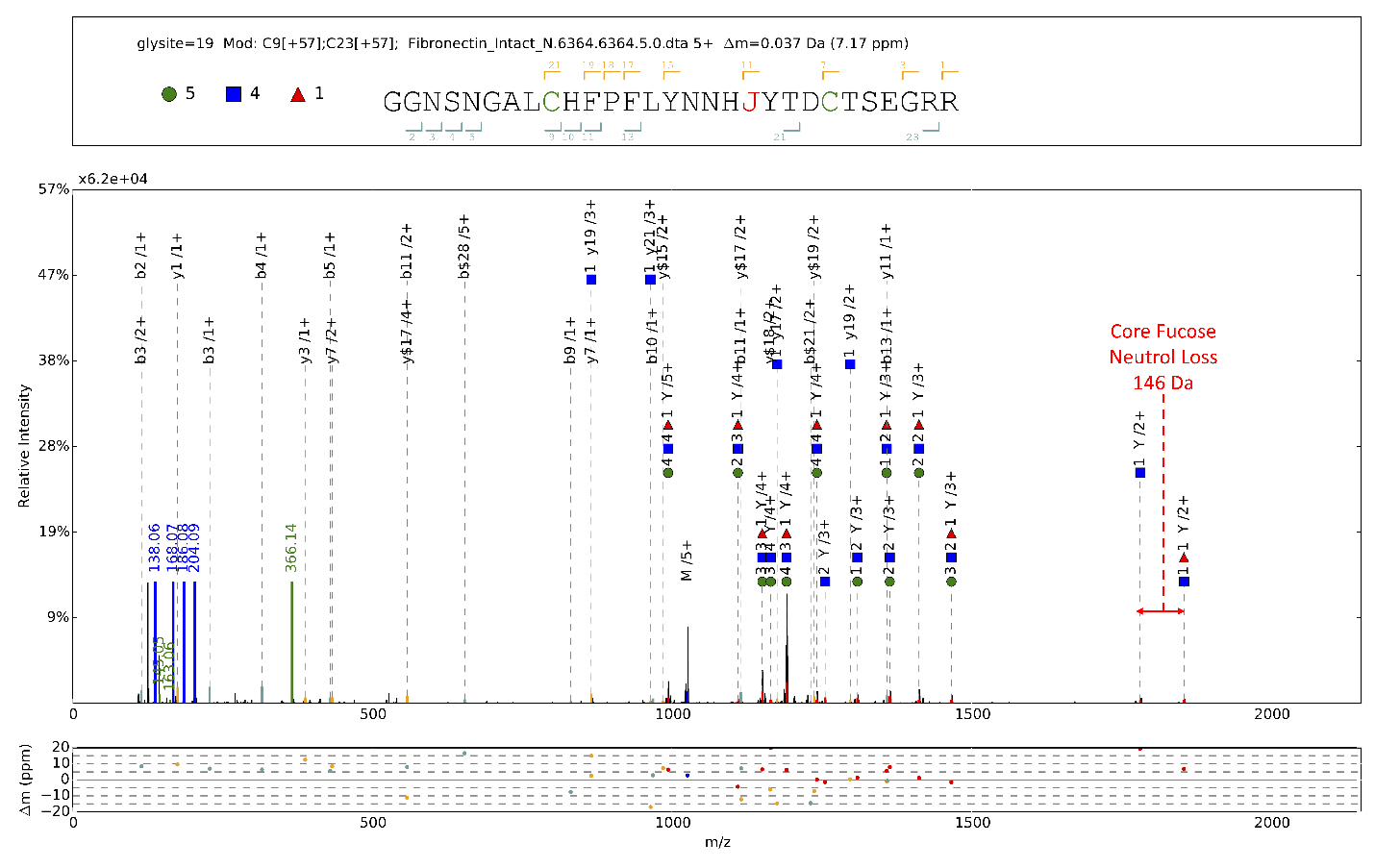
Figure S1. Tandem Mass spectra annotation of N-glycopeptide contains site 430 (J represent the site).


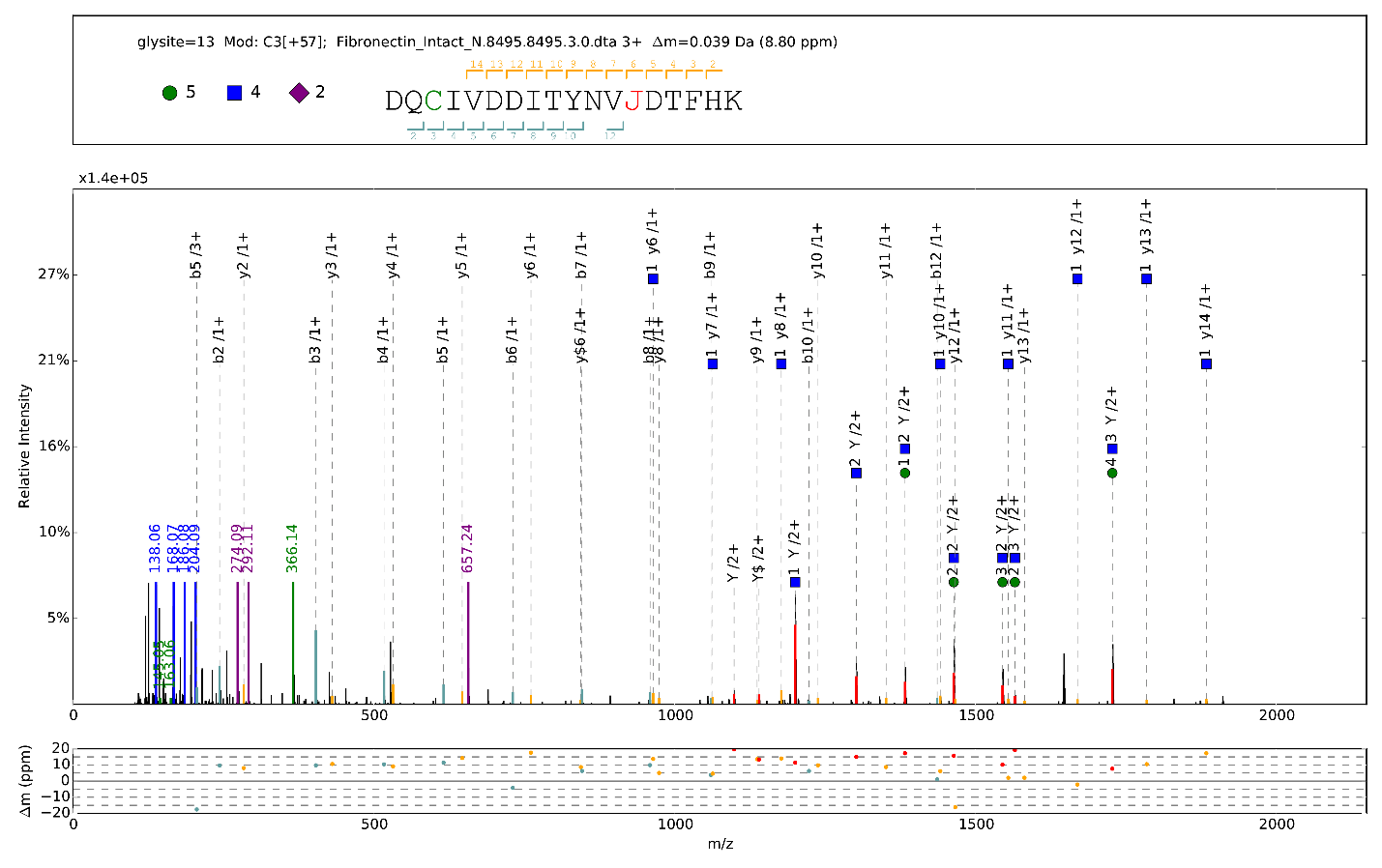


Figure S2. Tandem Mass spectra annotation of N-glycopeptide contains site 528 (J represent the site).


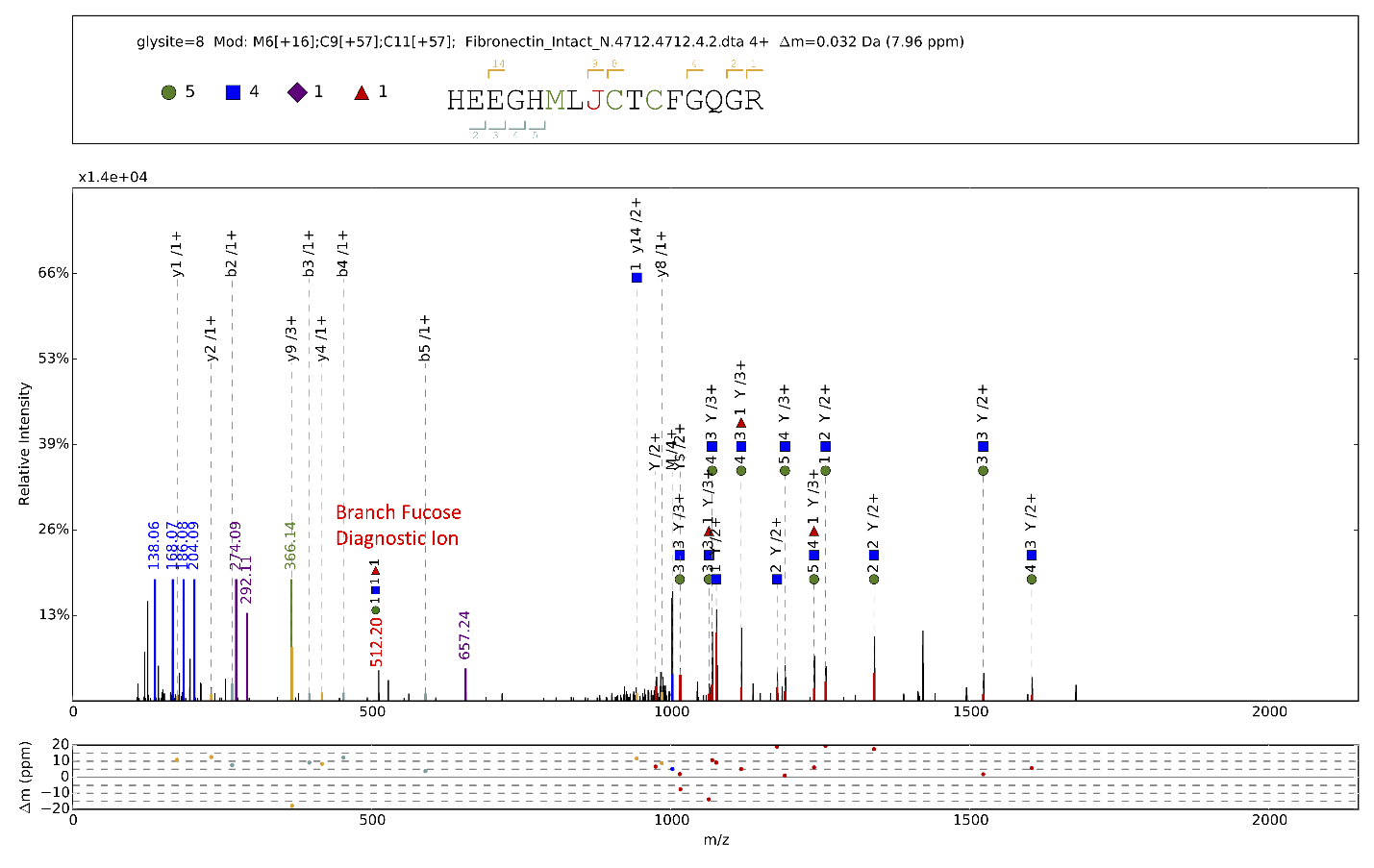
Figure S3. Tandem Mass spectra annotation of N-glycopeptide contains site 542 (J represent the site).


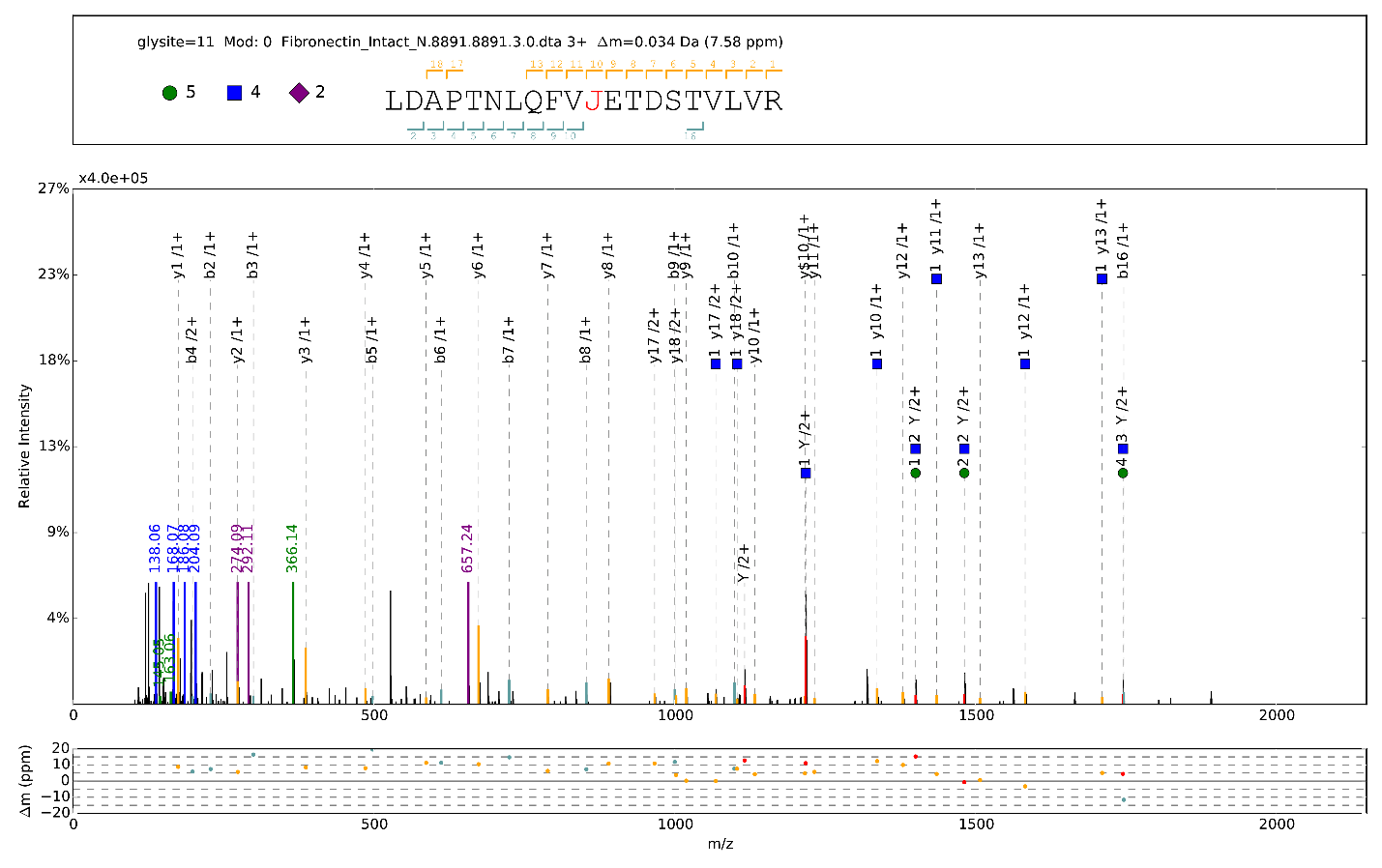
 Figure S4. Tandem Mass spectra annotation of N-glycopeptide contains site 1007 (J represent the site).


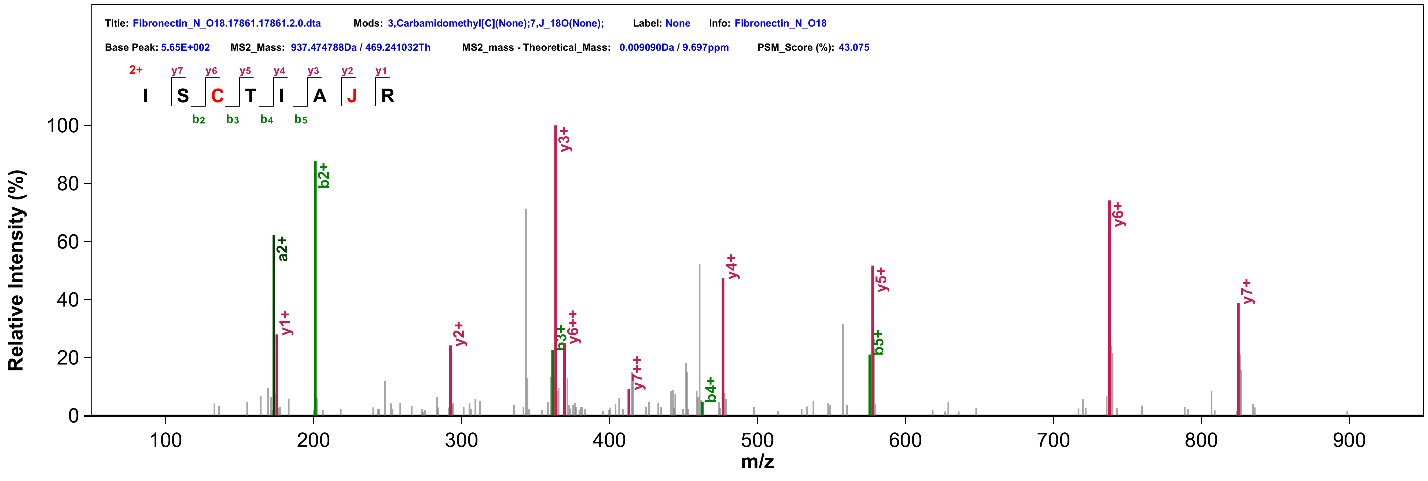
 Figure S5. Tandem Mass spectra annotation of ^18^O labeled N-glycosylation site 139 (J represent the site).


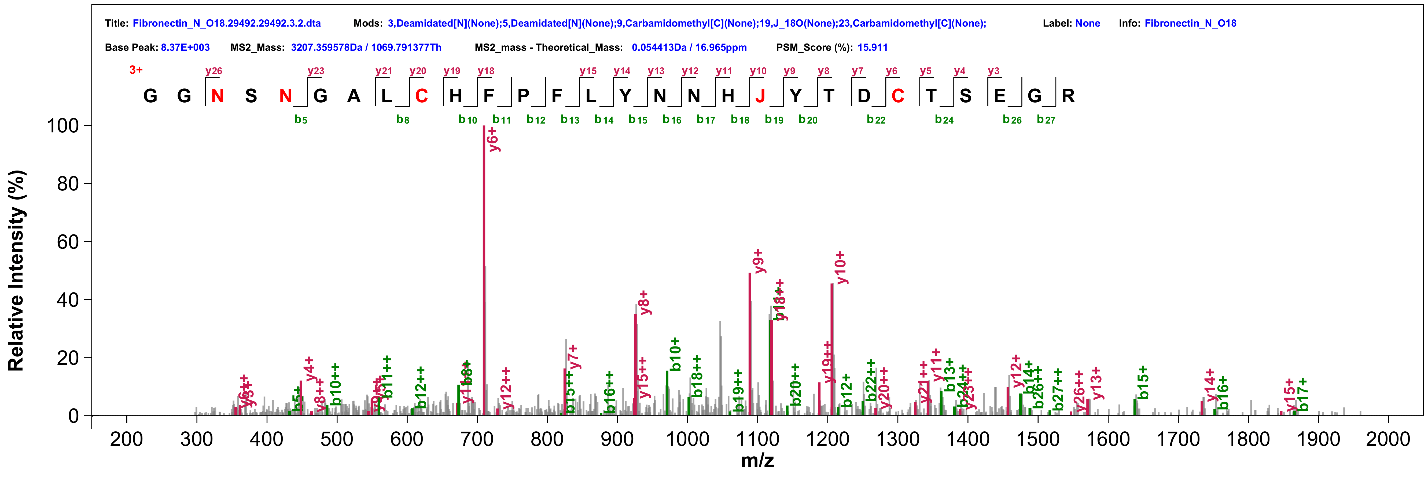
Figure S6 Tandem Mass spectra annotation of ^18^O labeled N-glycosylation site 430 (J represent the site).


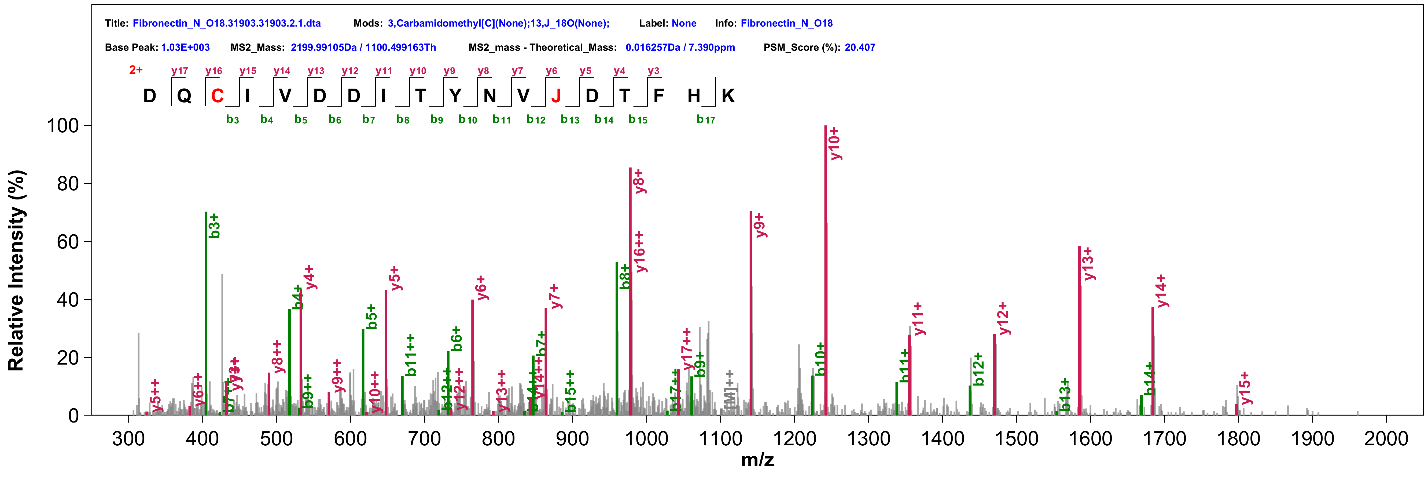


Figure S7 Tandem Mass spectra annotation of ^18^O labeled N-glycosylation site 528 (J represent the site).


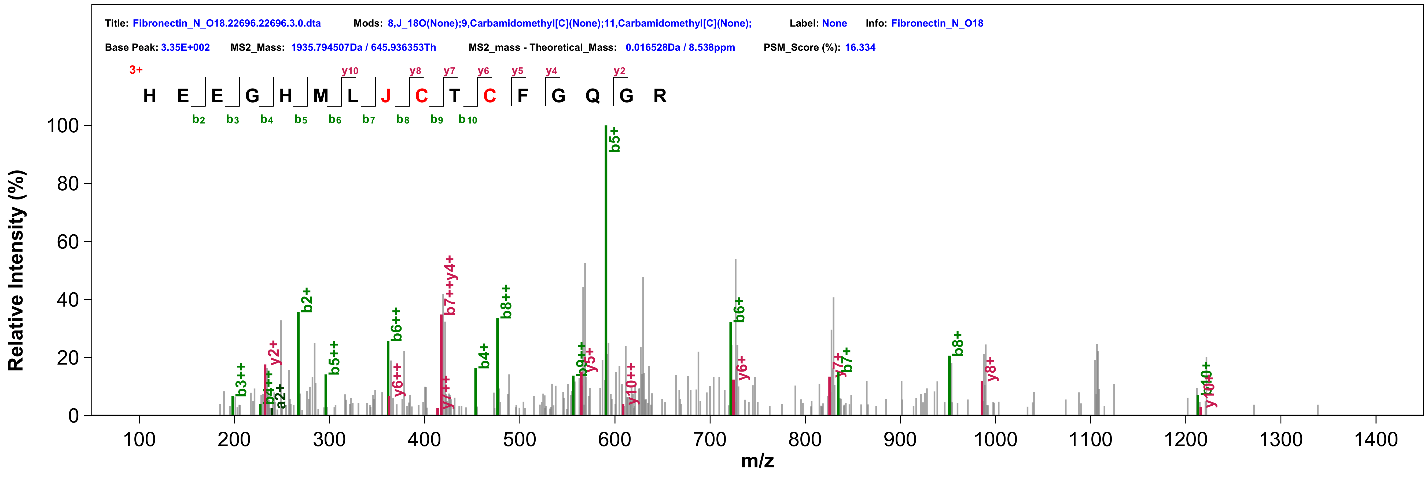


Figure S8 Tandem Mass spectra annotation of ^18^O labeled N-glycosylation site 542 (J represent the site).


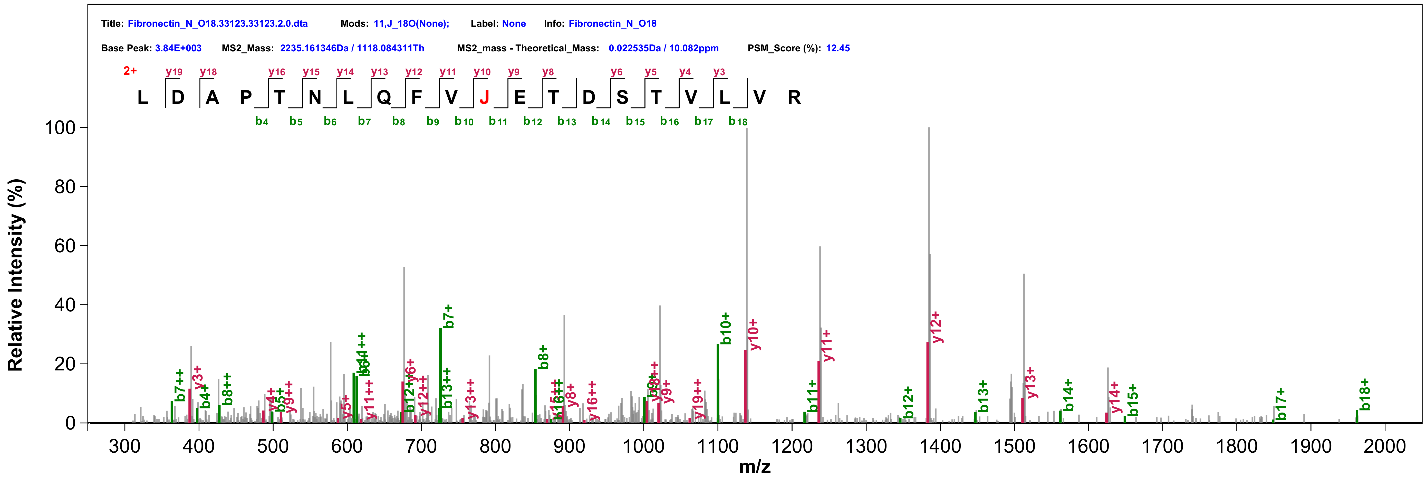


Figure S9 Tandem Mass spectra annotation of ^18^O labeled N-glycosylation site 1007 (J represent the site).


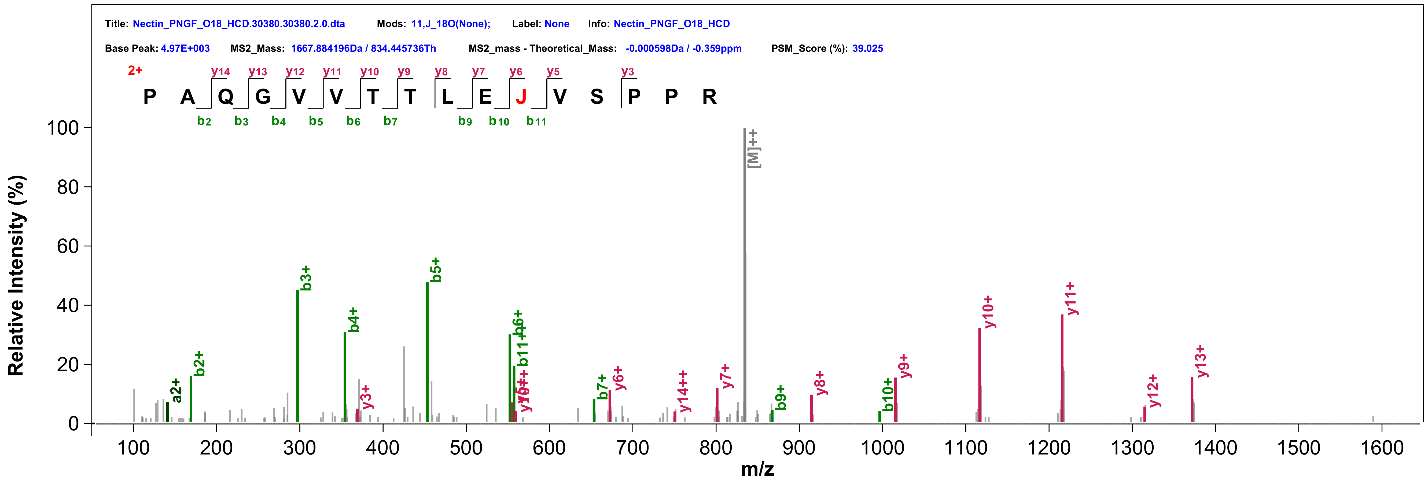


Figure S10 Tandem Mass spectra annotation of ^18^O labeled N-glycosylation site 1904 (J represent the site).


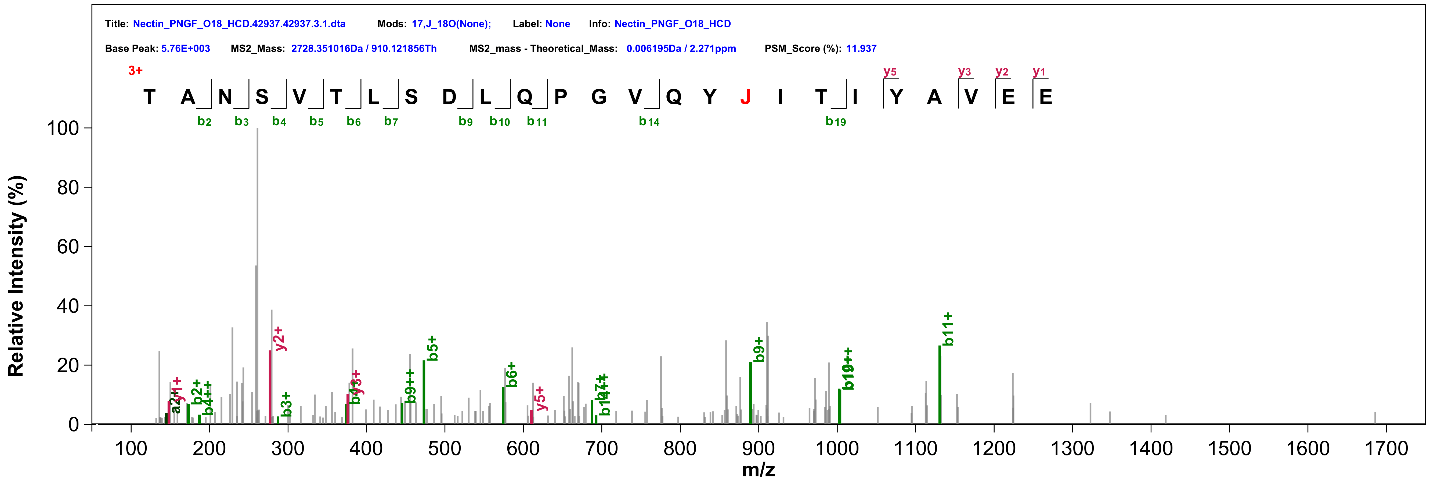


Figure S11 Tandem Mass spectra annotation of ^18^O labeled N-glycosylation site 877 (J represent the site).


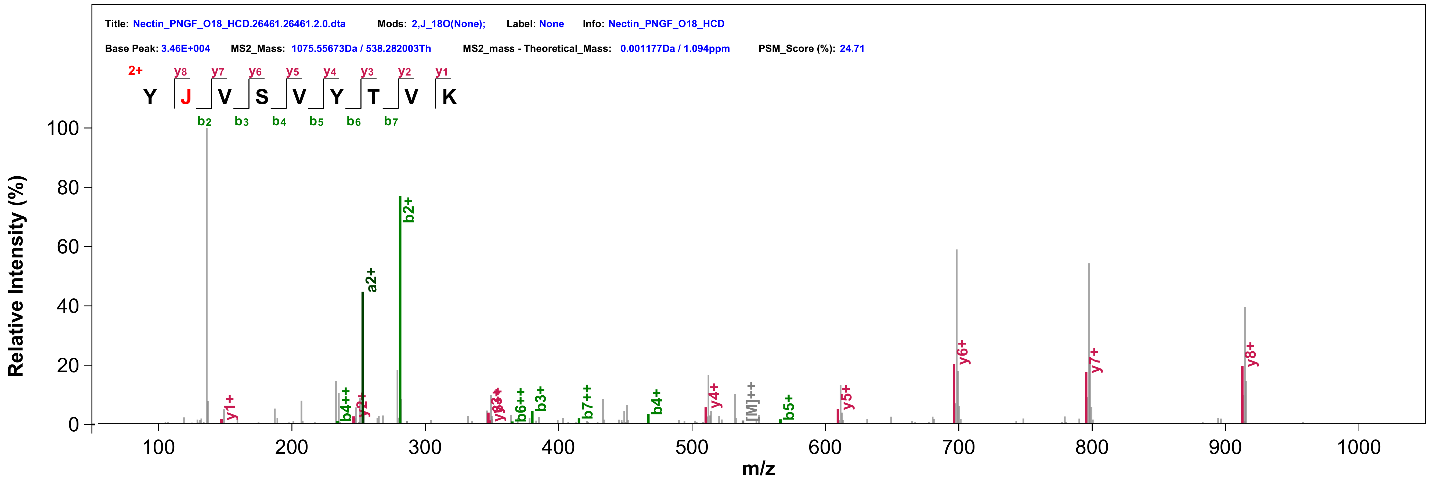


Figure S12 Tandem Mass spectra annotation of ^18^O labeled N-glycosylation site 1244 (J represent the site).


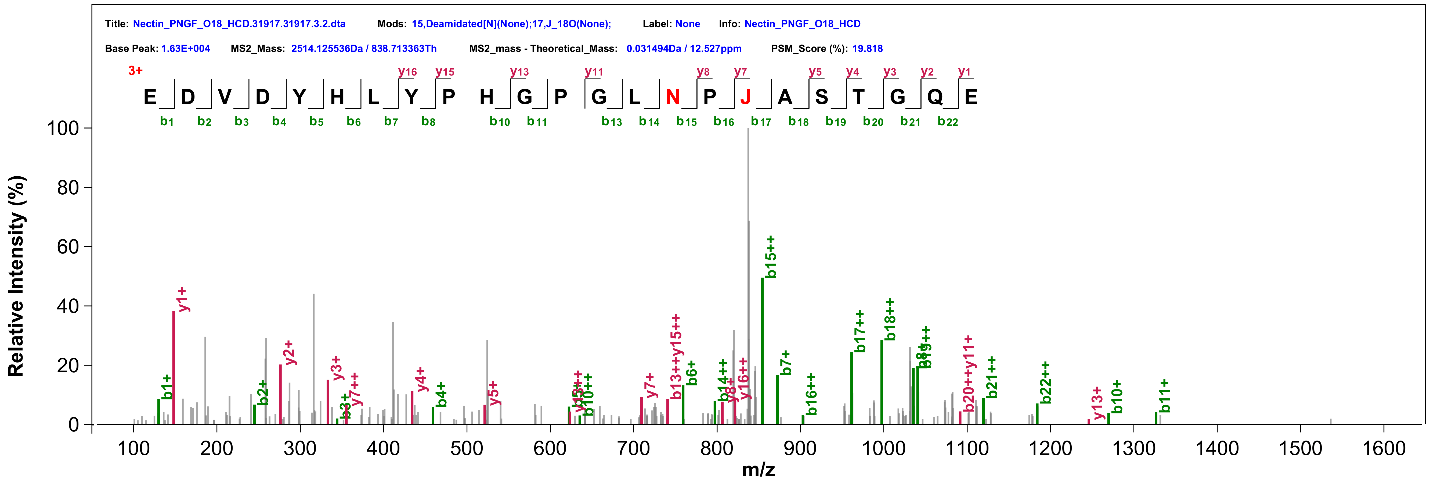


Figure S13 Tandem Mass spectra annotation of ^18^O labeled N-glycosylation site 2199 (J represent the site).


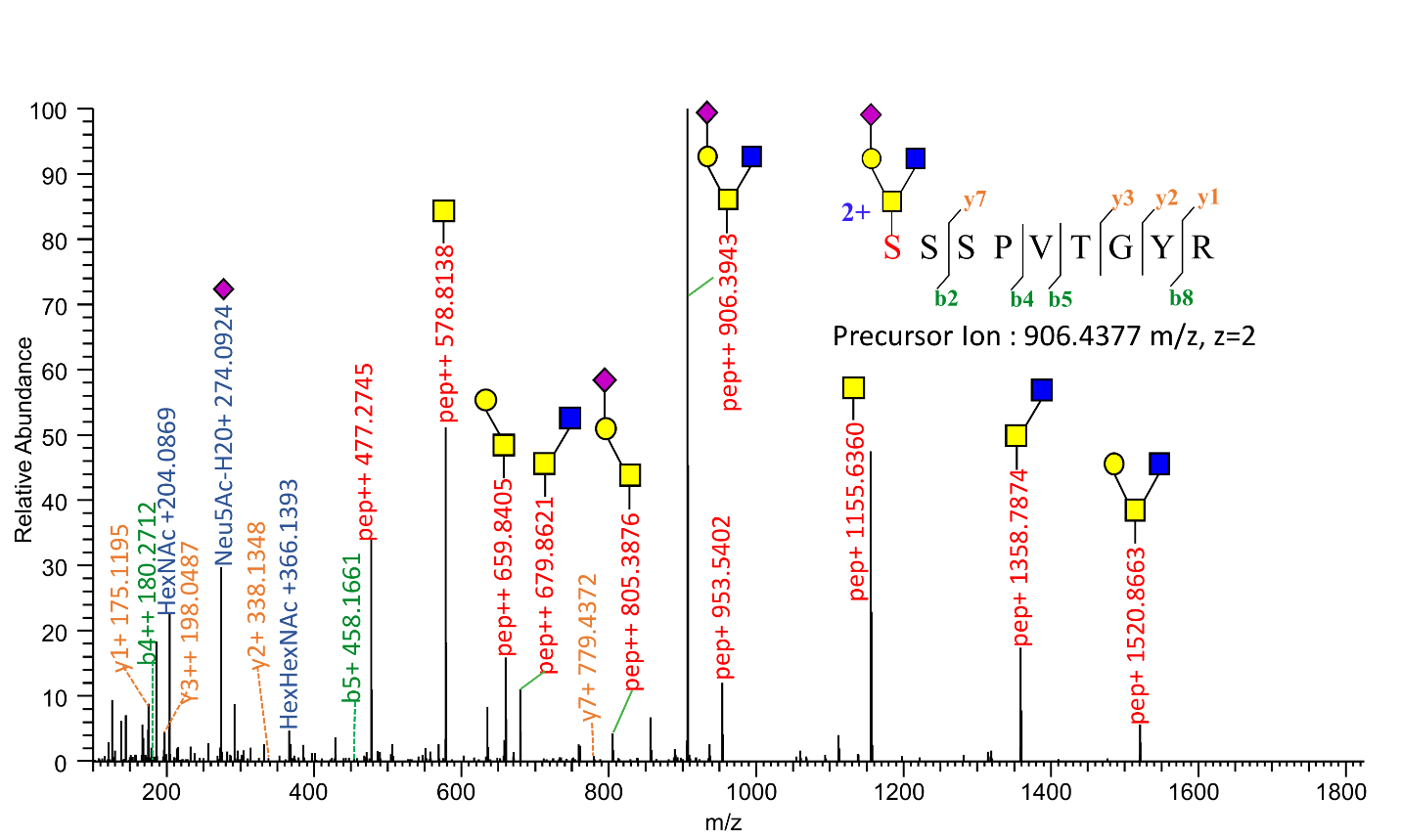


Figure S14. Tandem Mass spectra annotation of sialylated O-glycopeptide contains site Ser-1656.


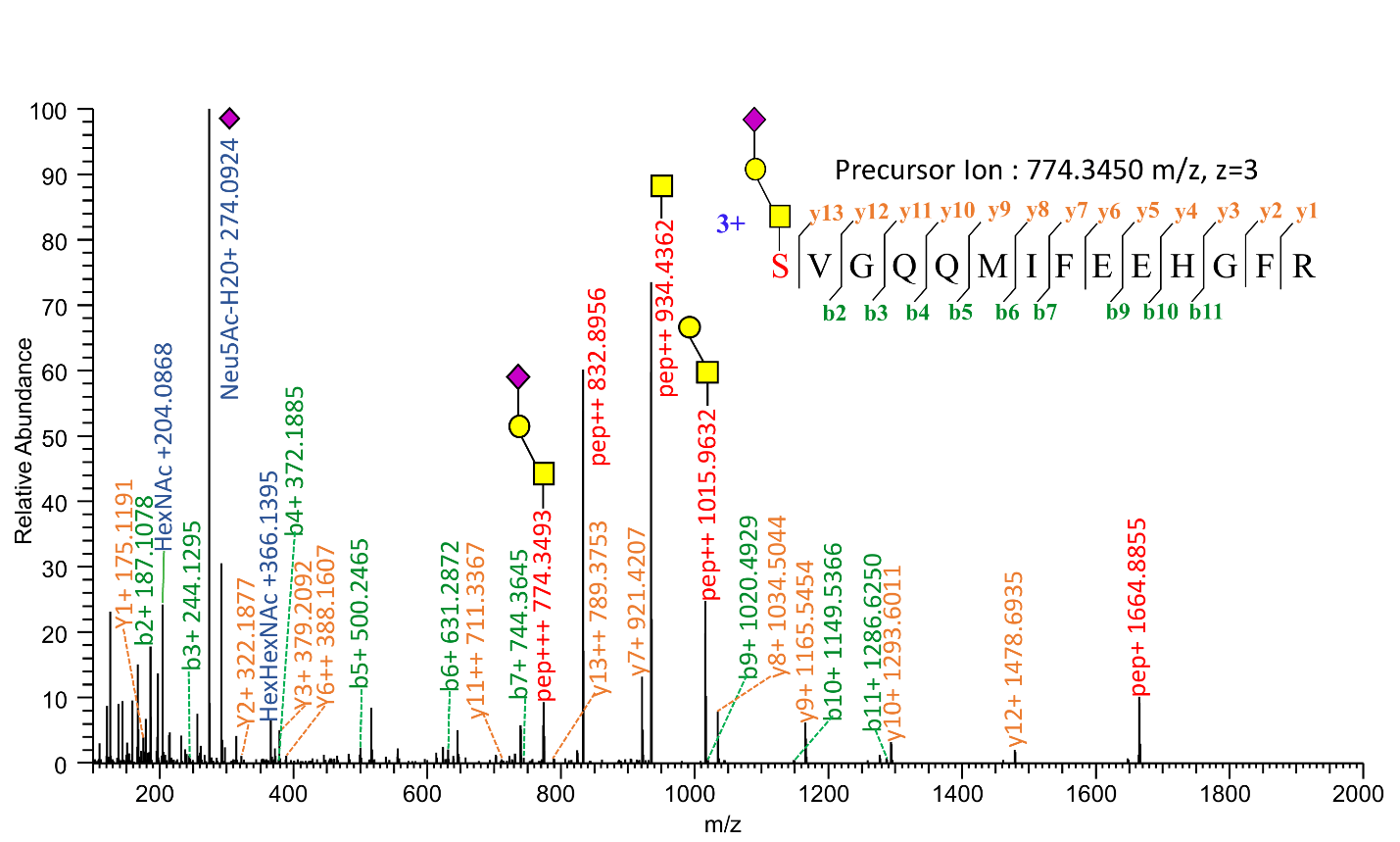
Figure S15. Tandem Mass spectra annotation of sialylated O-glycopeptide contains site Ser-2136.


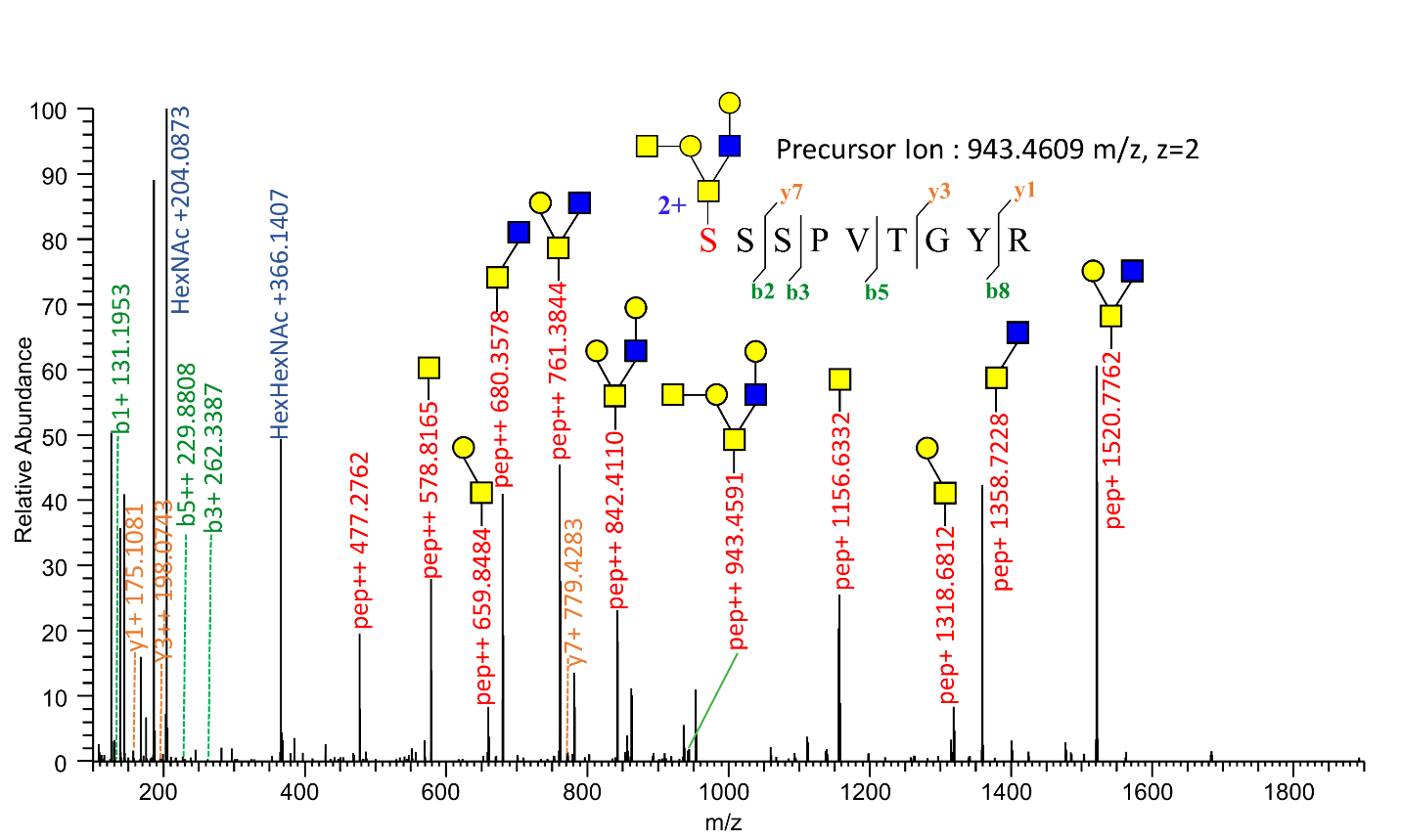


Figure S16. Tandem Mass spectra annotation of O-glycopeptide contains site Ser-1656. (multiple O-glycosites for peptides with repetitive sequences cannot be excluded)


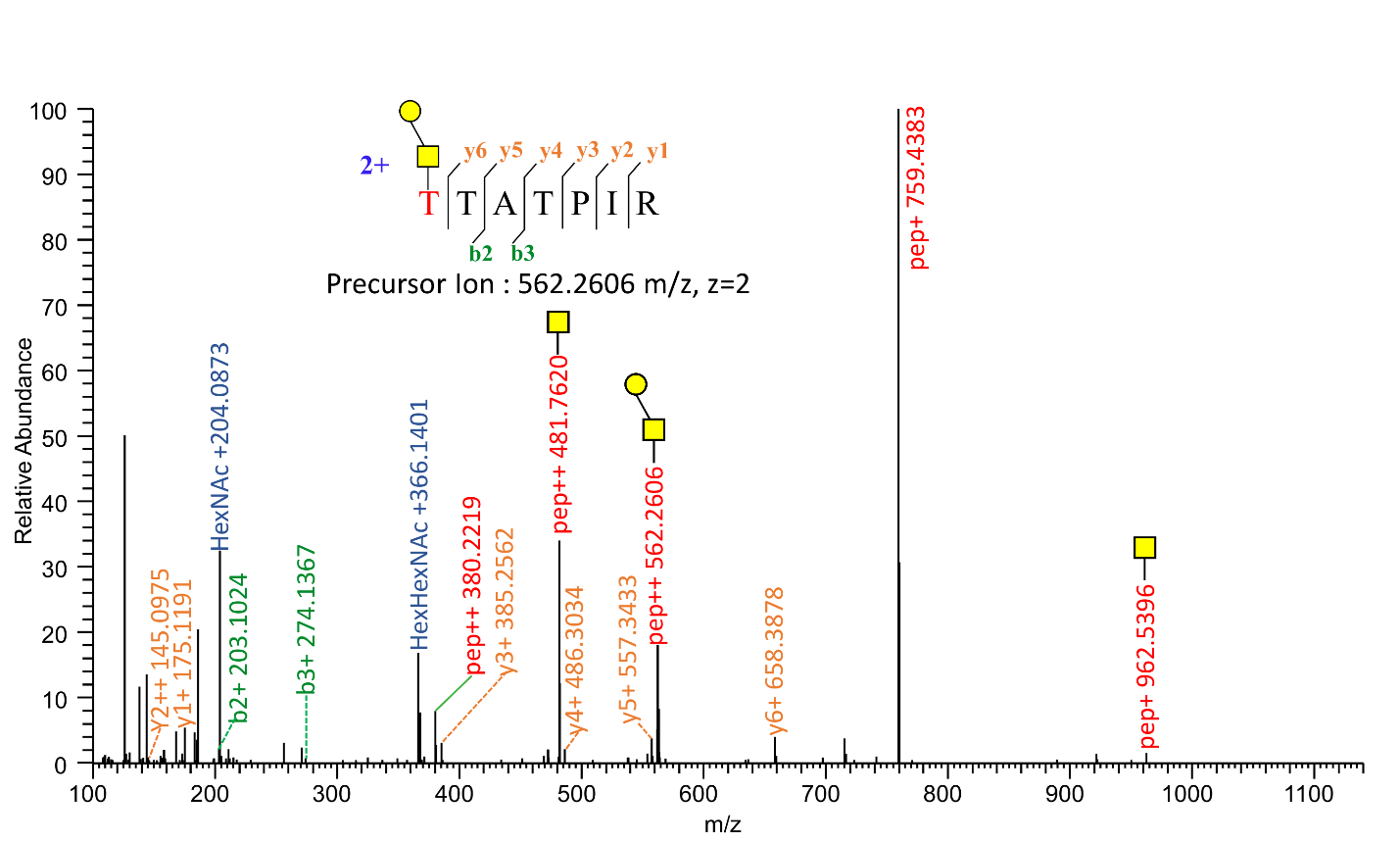
 Figure S17. Tandem Mass spectra annotation of O-glycopeptide contains site Thr-2155.


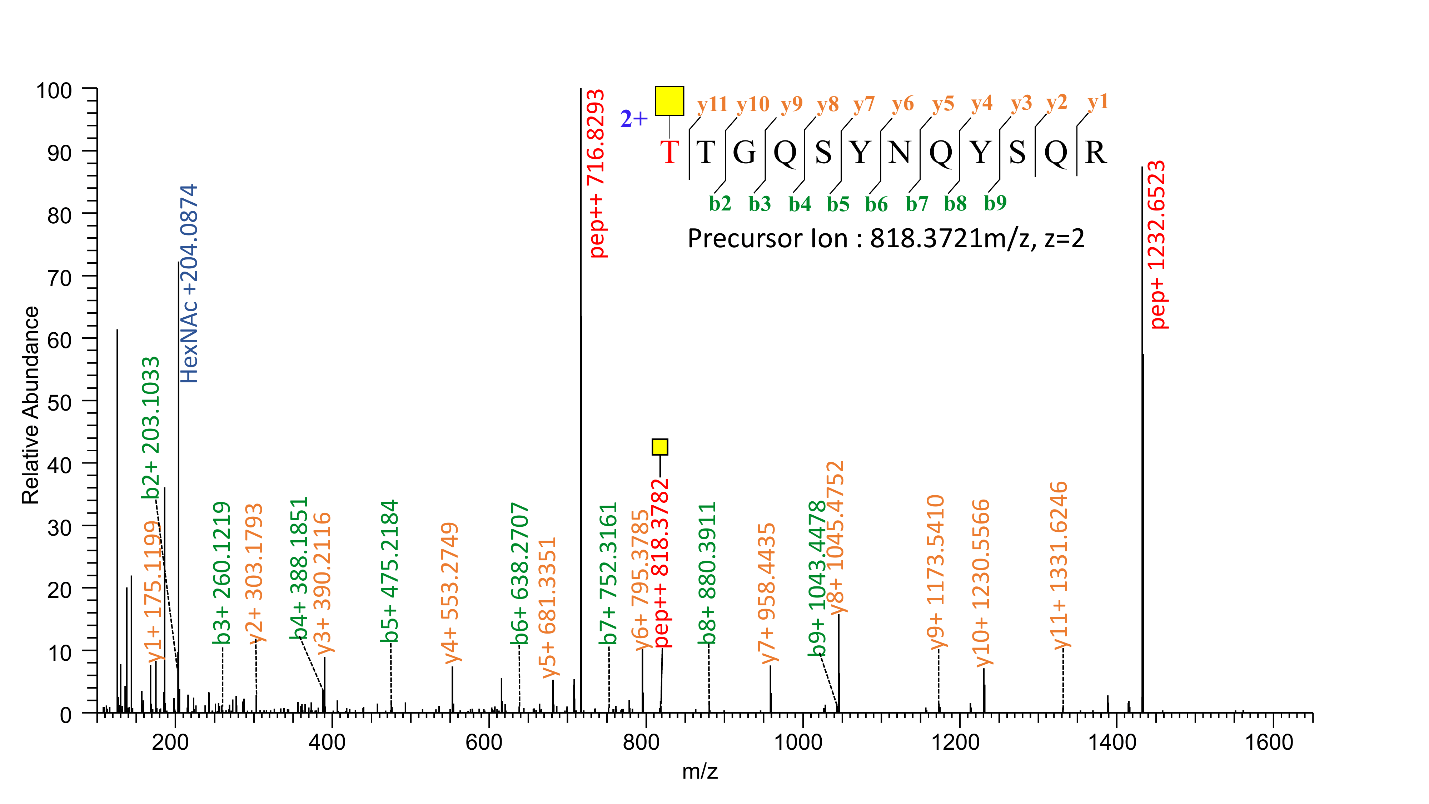
Figure S18. Tandem Mass spectra annotation of O-glycopeptide contains site Thr-2436.


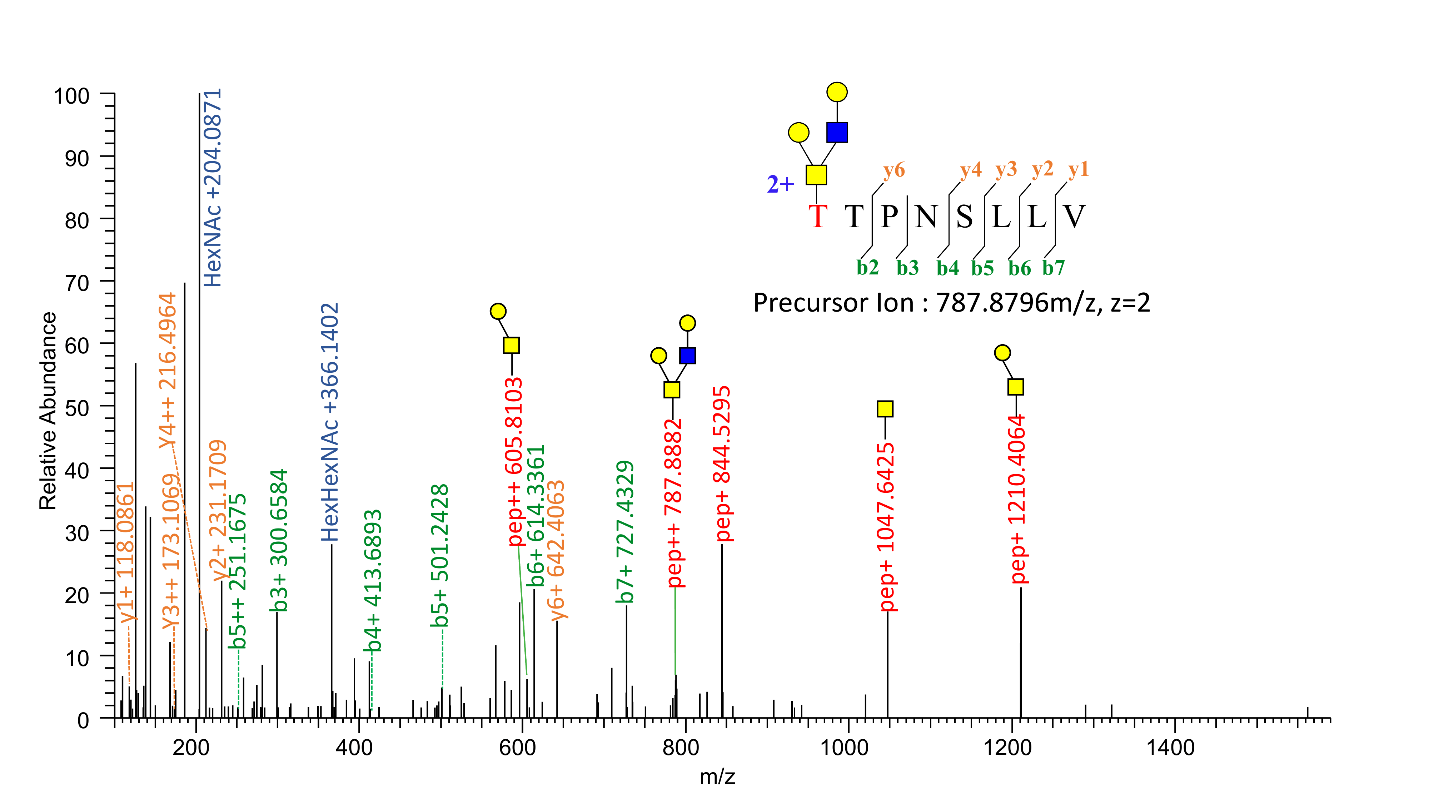


Figure S19. Tandem Mass spectra annotation of O-glycopeptide contains site Thr-2005. (multiple O-glycosites for peptides with repetitive sequences cannot be excluded)


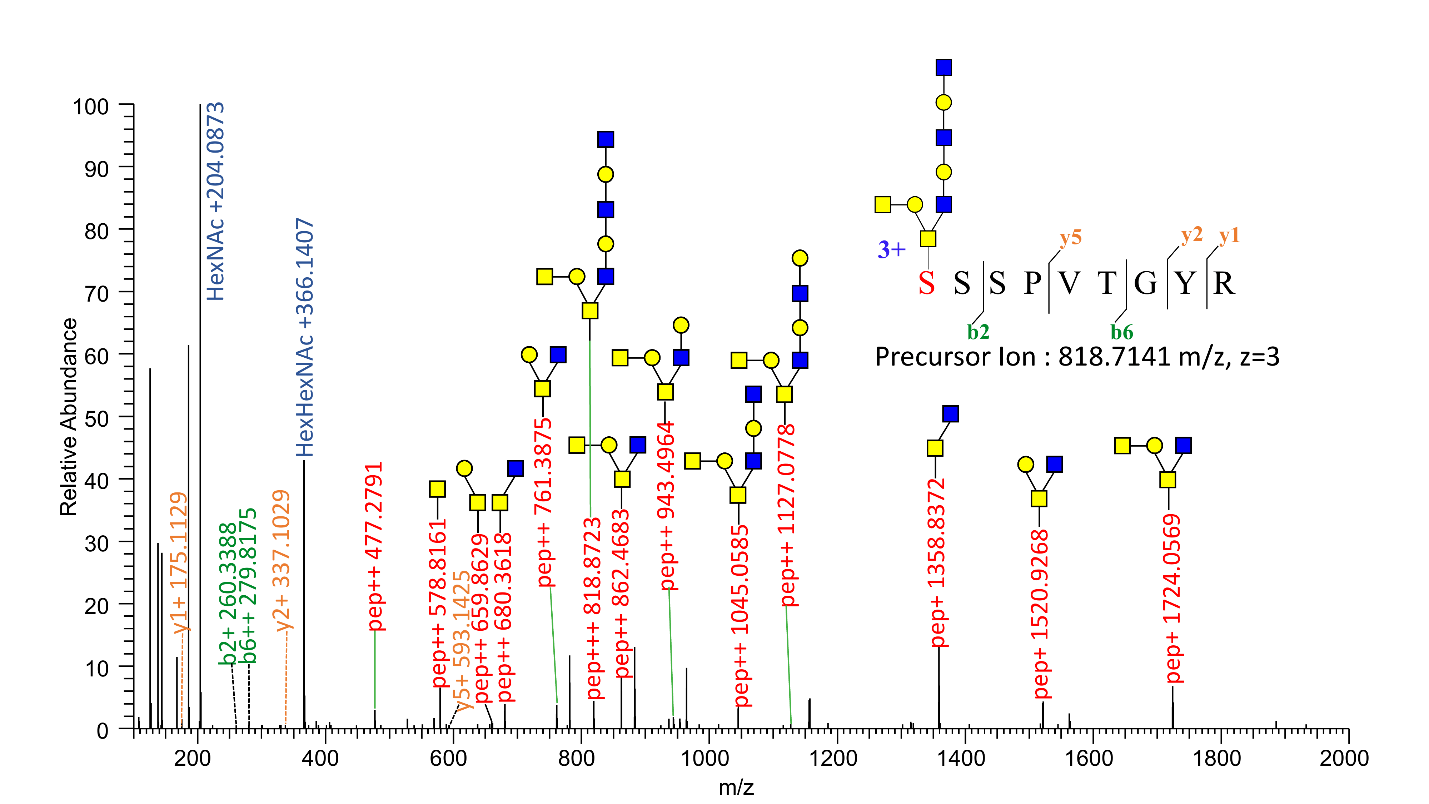


Figure S20. Tandem Mass spectra annotation of O-glycopeptide contains site Ser-1656 with compound 54. (multiple O-glycosites for peptides with repetitive sequences cannot be excluded)

Figure S21. Tandem Mass spectra annotation of O-glycopeptide contains site Ser-1656 with compound 59. (multiple O-glycosites for peptides with repetitive sequences cannot be excluded)
